# WNetAlign: fast and accurate spectra alignment using truncated Wasserstein distance and network simplex

**DOI:** 10.1093/bib/bbag247

**Published:** 2026-05-25

**Authors:** Justyna Król, Maria Bochenek, Sylwia Jopa, Krzysztof Kazimierczuk, Anna Gambin, Michał Piotr Startek

**Affiliations:** Faculty of Mathematics, Informatics and Mechanics, University of Warsaw, Banacha 2, 02-097 Warsaw, Poland; Faculty of Mathematics, Informatics and Mechanics, University of Warsaw, Banacha 2, 02-097 Warsaw, Poland; Centre of New Technologies, University of Warsaw, Banacha 2C, 02-097 Warsaw, Poland; Centre of New Technologies, University of Warsaw, Banacha 2C, 02-097 Warsaw, Poland; Faculty of Mathematics, Informatics and Mechanics, University of Warsaw, Banacha 2, 02-097 Warsaw, Poland; Faculty of Mathematics, Informatics and Mechanics, University of Warsaw, Banacha 2, 02-097 Warsaw, Poland

**Keywords:** nuclear magnetic resonance spectroscopy, liquid chromatography–mass spectrometry, spectra alignment, optimal transport, Wasserstein distance, network simplex algorithm

## Abstract

Liquid chromatography–mass spectrometry (LC–MS) and nuclear magnetic resonance (NMR) spectroscopy are complementary analytical techniques widely used in proteomics, metabolomics, and structural biology. Both generate high-dimensional, noisy spectra where overlapping peaks complicate interpretation. LC–MS relies on retention time (RT) separation before mass analysis, while multidimensional NMR spreads information across chemical-shift axes to reduce congestion. However, comparative or replicate experiments often introduce RT shifts in LC–MS or frequency shifts in NMR, hindering accurate matching of corresponding features. In some experiments, such as variable-temperature NMR, the shifts are intentionally triggered, and frequency tracking provides important information. In any case, a robust, scalable alignment across runs is critical for reliable compound identification, quantification, and structural analysis. We propose a truncated Wasserstein distance-based algorithm for aligning LC–MS and NMR spectra. By constraining maximum transport distance and formulating alignment as a minimum-cost flow problem solved via the Network Simplex algorithm, our method accelerates computation, suppresses spurious matches, and improves robustness to noise. On benchmark LC–MS datasets, it achieved 0.97 precision, 0.96 recall, and a 0.6-s runtime, outperforming OpenMS and DeepRTAlign tools. For NMR data, the algorithm proved effective in 2D, 4D, and even 7D analyses. The algorithm is implemented in wnetalign with supporting modules wnet and pylmcf, available on PyPI and GitHub under permissive licenses: https://github.com/michalsta/pylmcf, https://github.com/michalsta/wnet, https://github.com/michalsta/wnetalign.

## Introduction

Liquid chromatography–mass spectrometry (LC–MS) and nuclear magnetic resonance (NMR) spectroscopy are among the most widely used analytical techniques in chemistry, biology, and medicine. They provide complementary insights into the composition, structure, and dynamics of complex molecular systems.

While LC–MS offers high sensitivity and is particularly effective for compound detection and quantification in complex mixtures, NMR provides detailed information about molecular structure and dynamics. However, both techniques generate large, high-dimensional datasets that are often noisy, redundant, and computationally demanding to interpret.

A mass spectrometer measures the mass-to-charge ratio (m/z) of ions and presents the results as a mass spectrum. In such spectra, the intensity is plotted as a function of m/z.

In the NMR spectrum, each compound gives rise to a characteristic pattern of peaks, often sufficient to identify it. Unlike LC–MS, no reference is necessary—the molecular structure can be determined *ab initio*, at least in simple cases. For complex samples, such as macromolecules or mixtures, the overlap of spectral peaks hampers the analysis. To address this problem, various multidimensional NMR experiments were developed. Multidimensional NMR spreads the spectral information across two or more chemical shift axes, effectively adding new dimensions to the data. This reduces spectral congestion and improves the separation of overlapping signals, thereby enhancing interpretability and enabling the structural analysis of large biomolecules, such as proteins and nucleic acids. Moreover, the multidimensional experiments enable the observation of internuclear couplings via various interactions, boost sensitivity through indirect detection of nuclei resonating at low frequencies, and even allow the observation of directly undetectable multiple-quantum coherences.

In this context, it is instructive to compare NMR with another widely used technique in analytical chemistry: LC–MS. While fundamentally different in physical principles, both NMR and LC–MS share a similar analytical challenge: the need to resolve overlapping signals arising from complex mixtures. In LC–MS, this is achieved by chromatographic separation prior to mass analysis. Components of a sample are separated based on their interactions with the stationary phase inside a chromatography column, and elute at different RTs before being introduced into the mass spectrometer. The resulting spectra span 3D: mass-to-charge ratio, retention time (RT), and signal intensity.

Both multidimensional NMR and LC–MS address the problem of signal overlap by introducing additional dimensions that help distinguish between different chemical components. In both techniques, these higher-dimensional representations aim to disentangle the contributions of individual compounds and improve the specificity and interpretability of the data.

However, increasing the number of dimensions comes at a cost: the volume and complexity of the resulting datasets grow substantially. This, in turn, introduces significant challenges related to data processing—including signal extraction, noise reduction, alignment, and the identification of meaningful patterns within high-dimensional, often noisy, measurements. These issues are central across both modalities and have motivated the search for more effective strategies of spectral data interpretation in complex chemical and biological systems.

### Motivation and related research

Performing LC–MS experiments typically involves conducting multiple runs of the same or similar biological samples, either as technical or biological replicates. Technical replicates consist of repeated runs of the same sample to assess instrument reproducibility. Biological replicates, in contrast, capture biological variability by analyzing samples obtained under different conditions or at various time points. In both cases, analytes may elute at varying RTs and occasionally at slightly different mass-to-charge (m/z) values. While variation in m/z is usually minimal due to high mass resolution, RT variability can be substantial. These shifts may result from numerous factors, including instrumental drift, temperature fluctuations, column aging, ionization efficiency, variation in signal intensity, and random effects during sample collection or injection. Notably, such RT shifts can occur even in well-calibrated LC–MS systems operated within the same laboratory and on the same instrument [1].

Before further analysis, it is essential to reconcile these variations by aligning the spectra, i.e. mapping corresponding analytes across runs. While certain aspects of RT variability can be minimized experimentally, much of the alignment process relies on computational methods. LC–MS data alignment aims to produce a list of consensus features—groups of peaks, each representing the same chemical entity across multiple runs. In the case of pairwise alignment, consensus features are defined as matching peak pairs from two spectra; for more than two runs, they are sets of matched features ideally present in all spectra.

Numerous alignment algorithms have been proposed, many of which rely on a warping function—a transformation that models systematic RT distortions across runs. However, the ultimate goal remains the same: to accurately identify corresponding peaks in different spectra that represent the same analyte. Due to the high complexity of LC–MS data, only a few algorithms attempt to model this directly and robustly [2]. Furthermore, it is important to account for spurious peaks, which may arise from noise or sample-specific compounds not found in other spectra. These should remain unaligned to avoid introducing false matches.

A similar challenge is encountered in NMR spectroscopy, especially in comparative studies involving multiple multidimensional NMR spectra. In this setting, peak shifts across spectra are common and may be caused by environmental effects (e.g. pH or temperature differences), instrument variability, or specific sample interactions. As in LC–MS, these shifts hinder comparative and statistical analyses, as well as automated structure elucidation. Sometimes, however, they are introduced in a controlled manner and provide important information. For example, the temperature-induced shifts are probes of hydrogen bonding, solvent accessibility [3, 4], and structure changes [5]. Their non-linearities are connected to the presence of low-populated excited states of a protein [6, 7]. To measure these so-called temperature coefficients, effective peak-tracking algorithms have to be developed [8].

While alignment in 1D NMR is already a non-trivial task, multidimensional NMR alignment presents a significantly more complex problem. Misalignments can occur along multiple frequency dimensions simultaneously, and the direction and magnitude of the shifts may vary between dimensions and across peaks.

Among recent developments, optimal transport theory has emerged as a promising framework for addressing various spectral alignment problems. For instance, the Masserstein algorithm applied the 1D Wasserstein distance to deconvolve mass spectra, successfully separating overlapping isotopic envelopes and filtering out noise [9]. This idea was extended to NMR by the Magnetstein algorithm, which incorporated a denoising step tailored for 1D NMR spectra. However, both approaches remained restricted to univariate data.

In the context of LC–MS alignment, the Alignstein algorithm [10] applied the Sinkhorn approximation to compute optimal transport maps between spectra. Although it demonstrated a strong alignment performance, especially on noisy data, it suffered from computational inefficiency when applied to large-scale datasets.

Beyond optimal transport-based strategies, a broad range of LC–MS alignment methods have been developed and systematically benchmarked. A comparative evaluation presented in [2] examined commonly used tools such as OpenMS [11, 12], msInspect [13], SpecArray [14], XAlign [15], XCMS [16], and MZmine [17]. These tools represent a variety of approaches, including RT correction, peak matching heuristics, and statistical modeling. One of the benchmark datasets from that study was also used in the present work for evaluation and for comparison with the Alignstein algorithm. The corresponding results are provided in the Supplementary Material.

Several methods have been developed to tackle NMR alignment. While 1D NMR benefits from mature tools such as icoshift [18], CluPA [19], and Speaq [20], there remains a gap in robust and scalable alignment strategies for multidimensional spectra. In recent years, however, a number of promising developments have emerged.

In 2023, the GIPMA algorithm (Global Intensity-Guided Peak Matching and Alignment) was proposed for the alignment of 2D ^1^H-^13^C HSQC spectra in metabolomics. GIPMA uses intensity-weighted clustering to dynamically group peaks across multiple samples, achieving accurate alignment on over 80 real-world biological spectra with minimal parameter tuning [21].

Despite these advances, most approaches are still confined either to 1D or to 2D datasets or require manual intervention and problem-specific heuristics. At present, a general-purpose, automated, and scalable solution capable of aligning multidimensional NMR spectra—handling nonlinear distortions, partial peak overlap, and noisy data—remains an open challenge in the field.

In this paper, we propose a novel method for spectra alignment based on optimal transport theory. This framework addresses the problem of transferring mass between probability distributions with minimal cost, which we quantify using the truncated Wasserstein distance. To apply this concept in proteomics, spectra are modeled as discrete probability measures. This approach has previously been shown to be effective in 1D mass spectra analysis [9, 22], as well as in LC–MS alignment [10].

## Materials and methods

### Truncated Wasserstein distance

The Wasserstein distance provides a principled way to quantify dissimilarity between two probability measures by solving an optimal transport problem [23]. While its computation admits a closed-form solution in 1D, extending it to multidimensional data is substantially more challenging, as the optimal transport plan must be determined numerically.

Several approaches have been proposed to approximate the Wasserstein distance in higher dimensions, including entropy-regularized methods such as the Sinkhorn algorithm [24], sliced-Wasserstein distances based on 1D projections, and classical linear programming formulations [9, 22, 25]. However, these methods either scale poorly or provide only approximate solutions, and few of them allow efficient recovery of the transport plan itself.

In the context of spectra alignment, transporting all signal mass is neither necessary nor biologically justified. Therefore, we base our approach on the truncated Wasserstein distance, defined directly as an optimal transport problem 


(1) \begin{align*}& W_{\mathrm{tr}}(\rho,\nu) = \inf_{\gamma \in \Gamma(\rho,\nu)} \int \hat{d}(x,y)\,\mathrm{d}\gamma(x,y),\end{align*}

where $\Gamma (\rho ,\nu )$ denotes the set of admissible transport plans between probability measures $\rho $ and $\nu $, and $\hat{d}$ is the truncated cost function 


\begin{align*}& \hat{d}(x, y) = \begin{cases} d(x - y), & \text{if } d(x - y) \leq d_{\mathrm{max}}, \\ \delta_{\mathrm{max}}, & \text{if } d(x - y)> d_{\mathrm{max}}. \end{cases} \end{align*}

Here $d(\cdot )$ denotes a standard distance metric, such as the Euclidean norm or the $1$-norm. In the spectrometric setting, $x$ and $y$ represent the locations of spectral features (e.g. mass-to-charge ratios or chemical shift positions) in two spectra. The difference $x - y$ thus corresponds to a shift along the spectral axis. The truncation prevents transport between distant spectral features, restricting alignment to biologically plausible matches and improving the robustness and accuracy of the proposed method.

### WNetAlign: Wasserstein flow alignment

**Figure 1:**
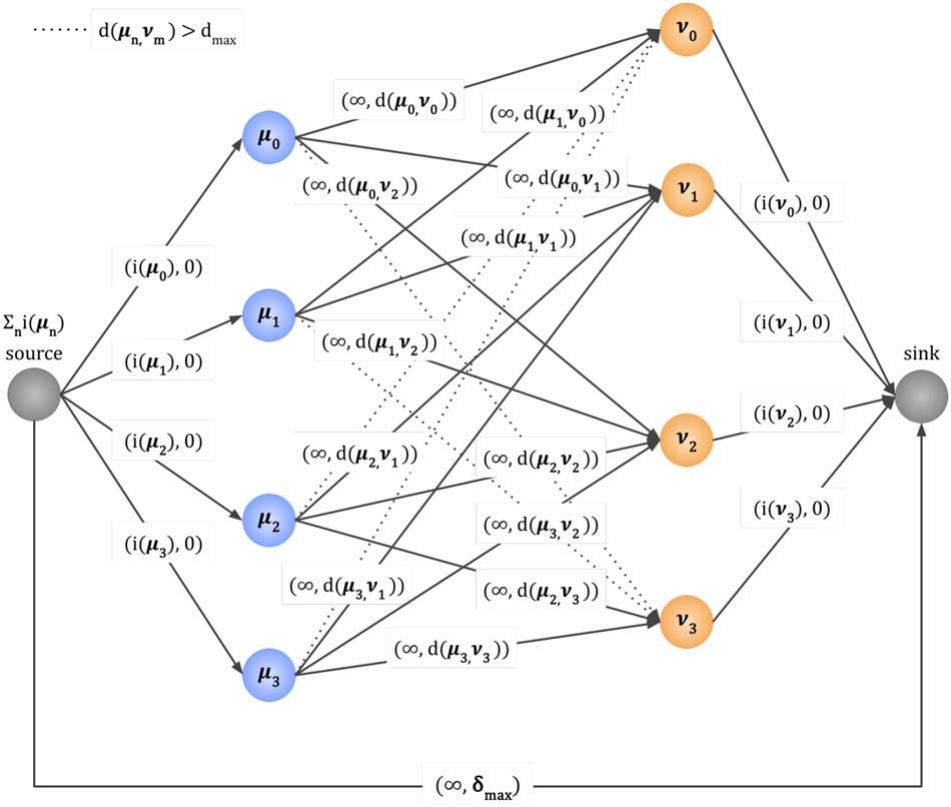
Flow network used to compute the optimal transport plan corresponding to the Truncated Wasserstein Distance between two spectra. The nodes $\mu _{j}$ represent the signals of the $\mu $ spectrum and the nodes $\nu _{k}$ the signals of the $\nu $ spectrum. Each edge is labeled with a pair of numbers ($f$, $c$), where $f$ is the flow capacity of the edge, and $c$ is the cost of transporting a unit of flow through the edge. For the edges connecting nodes $\mu _{j}$ to $\nu _{k}$, the cost of transporting a unit of flow is equal to the distance ($d$) between the signals $c = d(\mu _{j}, \nu _{k})$ while flow capacity is infinite. For incoming edges of $\mu _{j}$ nodes and outgoing edges of $\nu _{k}$ nodes, the flow capacity is equal to the peak’s signal intensity ($i(\mu _{j})$ and $i(\nu _{k})$, respectively), while transport cost equals zero. The edge joining source and sink nodes carries flow corresponding to unmatched signals.

The problem of calculation of optimal transport (with respect to the Truncated Wasserstein Distance) can be represented as an instance of the well-known Min-Cost-Flow problem [26], by constructing a special graph, as pictured in Fig. 1. The graph contains a node for every signal in each of the aligned spectra (blue and orange nodes, $\mu _{i}$ and $\nu _{i}$, respectively) and two special nodes: the *source* and the *sink* node. Next, we connect the source node and the nodes from the first spectrum (the blue ones) with edges, each with capacity $i(\mu _{i})$, i.e. the signal intensity of the $\mu _{i}$ peak, and zero cost. Similarly, we connect the (orange) nodes corresponding to the second spectrum to the sink. Next, for each pair of nodes in respective spectra, we join them with a “matching” edge of infinite capacity, and cost equal to the distance between the nodes. We also add a special “mismatch” edge with infinite capacity and cost equal to $\delta _{\mathrm{max}}$, which is a parameter of the algorithm. Last, it can be easily shown that “matching” edges with cost greater than $\delta _{\mathrm{max}}$ will never carry any flow, and can be safely dropped from the graph, without affecting the result (represented by dotted lines in Fig. 1). This has the additional benefit of speeding up the algorithm, because it will usually cause the graph to be sparse rather than dense. Furthermore, it also represents the expected behavior of the alignment: it represents the maximum distance of signal shifts which we expect in the spectra, and prevents signals that lie unreasonably far from one another from being matched.

Next, unlike previous approaches in spectrometry [9, 10, 22, 25], which constructed similar flow networks but ultimately diverged from a pure MCF formulation and were forced to use generic linear programming solvers, we use the Network Simplex algorithm [27] to compute the min-cost-flow in the constructed graph, resulting in a dramatic speedup. Although Network Simplex has an exponential theoretical worst-case runtime, in practice, it exhibits excellent performance—better than the best-known polynomial-time algorithms [28]. For reasonable values of $\delta _{\mathrm{max}}$, the alignment graph is typically sparse in practice (the number of edges is proportional to the number of signals, multiplied by a constant density factor). As a result, the observed runtime is nearly linear with respect to the number of signals in each spectrum.

### PXD000484 liquid chromatographymass spectrometry dataset

The algorithm was evaluated using the PXD000484 dataset from the PRIDE repository. This dataset consists of 56 breast cancer tissue samples, each obtained from a different patient. The samples were microdissected, sonicated, and subjected to in-solution digestion. Mass spectrometry analysis was performed using an LTQ-Orbitrap instrument coupled with a nano-LC system, generating Thermo.RAW files as output [29].

All further data preprocessing was carried out by the authors of this paper using the OpenMS 3.3.0 TOPP tools. All spectra were first converted to the.mzML format using the FileConverter tool, followed by feature detection using the FeatureFinderCentroided function. The process of establishing ground truth for alignment is described in detail in Section 1.1 of the Supplementary Materials.

### Alignment of the liquid chromatographymass spectrometry dataset

In LC–MS data, RT shifts are typically much larger in magnitude than shifts observed in the m/z dimension, which are generally highly stable, with deviations limited to small instrument-specific mass errors. When aligning spectra, these differences in scale must be taken into account. If uncorrected, the substantial RT shifts can overshadow the comparatively small m/z deviations, leading to suboptimal alignments. To address this imbalance, it is necessary to rescale the 2D so that RT and m/z shifts become comparable.

To account for the differing magnitudes of variation in RT and m/z, we express the alignment thresholds in terms of two interpretable parameters, max_mz_shift and max_rt_shift, which define the maximum expected shifts in each dimension. The m/z axis is rescaled by the ratio $\frac{\mathtt{max}{\_}\mathtt{rt}{\_}\mathtt{shift}}{\mathtt{max}{\_}\mathtt{mz}{\_}\mathtt{shift}}$, thereby ensuring that RT and m/z shifts are comparable in scale. After this normalization step, both $d_{\mathrm{max}}$ and $\delta _{\mathrm{max}}$ are set to max_rt_shift, providing a unified distance threshold for alignment across dimensions. Consequently, features can only be matched to those lying within the predefined maximum shifts in each dimension.

After this process, the spectra are aligned using the proposed algorithm. Spectra should be in centroid mode, where each feature is represented by a single point defined by its m/z, RT, and intensity. The alignment process then produces a list of consensus features, i.e. pairs of signals from aligned spectra corresponding to the same analyte.

To construct a ground truth reference for feature alignment, we annotated the raw mass spectrometry files using MaxQuant. The ground truth was then defined as the set of feature pairs that were annotated with the same high-confidence peptide sequence. The details are provided in Section 1.1 of Supplementary materials.

**Figure 2:**
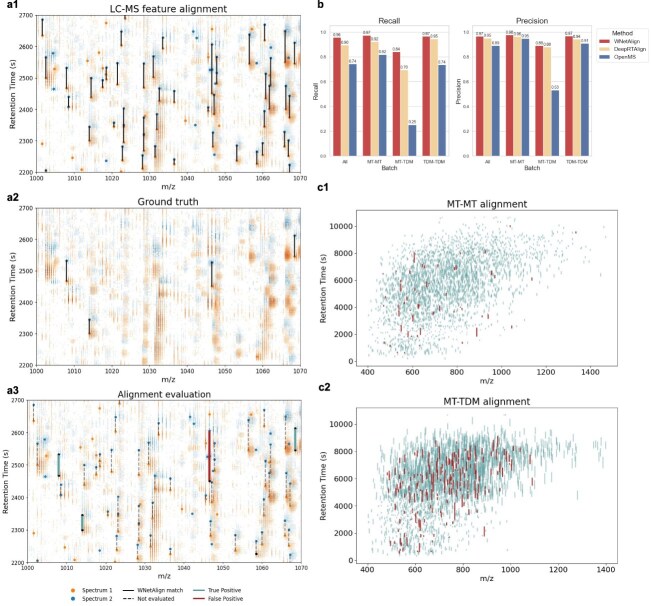
(a) Process of evaluation. In each panel, blue and orange represent aligned spectra. For clarity, only a small region of the spectra is shown. Large dots indicate features detected by OpenMS. (a1) Lines connect features aligned by the WNetAlign algorithm. (a2) Large dots indicate features with reliable peptide identifications. Lines connect features sharing the same peptide identification; these connections define the ground truth. (a3) Large dots represent all OpenMS-detected features, with black dots marking those with reliable peptide identifications. WNetAlign alignments are shown in blue if correct (true positives) and in red if incorrect (false positives), as defined in the Results section. Matches connected by dashed lines are excluded from evaluation due to insufficient reliable peptide identifications. (b) Evaluation results and comparison with DeepRTAlign and OpenMS. The PXD000484 dataset consists of two batches: MT and TDM. The performance of all algorithms varies between these batches. When aligning spectra across different batches, the WNetAlign algorithm maintains reasonable performance, DeepRTAlign performs slightly worse, while OpenMS fails to align reliably, achieving a recall of <0.3. Alignment results are significantly improved when spectra from the same batch are compared. Across all alignment scenarios, the WNetAlign algorithm consistently outperformed both DeepRTAlign and OpenMS. (c) Examples of alignment evaluation on the MT–MT pair of spectra and the MT–TDM pair of spectra. The lines connect matched features in both spectra. True positive matches are highlighted in blue, while false positive matches are highlighted in red. Longer lines correspond to greater RT shifts between spectra.

As proposed by Lange *et al*. [2], the primary metrics for evaluating LC–MS alignment methods are *recall* and *precision*, defined as $\mathrm{Recall} = \frac{\mathrm{TP}}{\mathrm{TP} + \mathrm{FN}} \text{ and Precision} = \frac{\mathrm{TP}}{\mathrm{TP} + \mathrm{FP}} $, where TP (true positives) are aligned pairs of features that were both assigned to the same peptide, FN (false negatives) are missed alignments that should have been detected, TP+FN is then equal to the number of feature matches in the ground truth. FP (false positives) are incorrect matches. In this context, the input to the alignment algorithm is the complete list of features detected by OpenMS, of which only a small subset has confident peptide identifications. A match is considered incorrect under two conditions: (i) the matched features have different peptide identifications, or (ii) one feature in the matched pair lacks a peptide identification, while the other is identified and present in the ground truth, indicating that it should instead be aligned to a feature with the same peptide identification. The procedure of evaluation is shown in Fig. 2.

### 2D $^{1}$H-$^{15}$N HSQC nuclear magnetic resonance dataset

**Figure 3:**
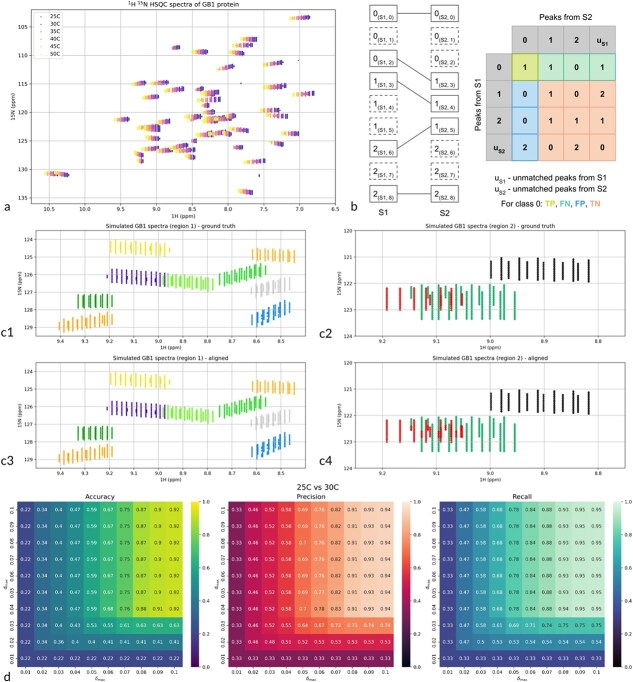
Evaluation on the NMR dataset. The GB1 1H 15N HSQC spectra measured in the different temperatures (a). Definition of performance metrics taking into account unmatched peaks from both spectra (b). To assess the quality of the chain alignment (Section 2.6), we clustered the first spectrum in a series ($25^{\circ }$C) using the K-Means algorithm. We then colored the peaks depending on the cluster of the starting point of the chain (c3 and c4) to assess how well the WNetAlign algorithm reconstructs the original cluster membership (c1 and c2). Performance evaluation for the $d_{\mathrm{max}}$ and $\delta _{\mathrm{max}}$ parameters shown for alignment of 25C and 30C spectra. Reference the main text for more details.

The algorithm was evaluated on the series of 2D $^{1}$H-$^{15}$N HSQC spectra of $^{15}$N-labeled GB1 protein (Immunoglobulin G-binding protein G, IgG-binding protein G) measured at different temperatures: $25^{\circ }C, 30^{\circ }C, 35^{\circ }C, 40^{\circ }C, 45^{\circ }C$, and $50^{\circ }C$ (Fig. 3).

The 1 mM GB1 protein sample in 10%/90% $\mathrm{D}_{2}\mathrm{O}$/H$_{2}$O, 20 mM sodium phosphate, pH 7.0 was purchased from Giotto Biotech). We used the peak assignment from the Biological Magnetic Resonance Bank (entry 7280) [30, 31].

We acquired the spectra on a 600 MHz DirectDrive2 Agilent spectrometer equipped with a room-temperature HCN probe with XYZ gradients. We used the gNhsqc pulse sequence from Biopack (VnmrJ 4.2 software) and the following parameters for $^{1}$H and $^{15}$N dimensions, respectively: spectral width $12\,019\times 2100$, number of complex sampling points $1024\times 64$, pulse width 8.125 $\mu $s and 42.175 $\mu $s. We processed spectra in nmrPipe [32] using zero-filling to $2048\times 128$ points, boxcar low-pass solvent filter, and 90$^{\circ }$ shifted sinebell apodization in both dimensions.

To reduce noise, negative intensities were discarded, and only peaks with intensities >10% of the maximum were retained. This threshold preserved between $36.73\%$ and $60.62\%$ of the original signal (Fig. S6).

### Chain alignment for series of nuclear magnetic resonance spectra

Pairwise alignments were performed between consecutive spectra in the temperature series, with all spectra normalized prior to alignment. For each peak $\mu _{i}$ in the first spectrum (Fig. 1), the principal match was defined as the peak $\nu _{j}$ in the subsequent spectrum receiving the largest flow from $\mu _{i}$ (Fig. 1). These maximal-flow correspondences were concatenated across the series to generate chain alignments from $25^{\circ }C$ through $50^{\circ }C$.

### Validation of WNetAlign on multidimensional nuclear magnetic resonance data

The proposed algorithm can be applied to NMR data of any dimensionality without the need for additional computations. However, to assess its quality, we require ground truth. For this reason, we introduce synthetic data, as described below.

#### 2D $^{1}$H-$^{15}$N HSQC nuclear magnetic resonance dataset

To establish ground truth for evaluating the algorithm, we generated a series of synthetic spectra replicating the original GB1 $^{1}$H-$^{15}$N HSQC spectra series using the procedure described in Section 2.2.1 of Supplementary materials. Moreover, to verify that performance results were not sample-dependent, the process of generating synthetic spectra was repeated 1000 times (Section 2.2.3 of Supplementary materials).

#### 4D CCNOESY (aliphatic) spectrum

To test scalability, we applied the algorithm to the 4D CCNOESY (aliphatic) spectrum of the SH3 domain of growth arrest-specific protein 7 (GAS7) (PDB structure ID: 2LX7) published as part of the 100-protein NMR spectra dataset [33]. A corresponding replicate spectrum was generated by randomly shifting the signals of the original spectrum as described in Section 3 of the Supplementary materials.

#### Hypothetical 7D nuclear magnetic resonance spectra

To further demonstrate robustness in high dimensions, we utilized 7D HN-N-CO-CA-CB-HA-HB NMR peak lists of 17 proteins published by Romero *et al*. [34]. The proteins’ BMRB entry IDs are provided in Table S4. In practice, the hypothetical 7D spectra were derived from combinations of peaks from 2D–4D spectra as described in [34].

Peaks with missing chemical shifts in at least 1D were discarded, resulting in 17 spectra (S4) with amino acid distribution shown in Fig. S32. Since the hypothetical spectra were constructed from peak lists, they did not include signal intensities; thus, all peaks were assigned unit intensity.

Corresponding replicate spectra were generated for each protein by shifting signals of the original spectrum according to the methodology from Section 4 of the Supplementary materials.

### Extended performance metrics for nuclear magnetic resonance alignment

To evaluate WNetAlign’s performance, we extended the definition of standard performance metrics to account for the peaks in each spectrum that were not matched. Each spectrum in the series of simulated 2D spectra has the same number of peaks in each cluster. The same is true for the 4D spectra. Similarly, for each 7D spectrum, the corresponding simulated 7D spectrum has the same number of peaks coming from the amino acid group.

Let us consider the matching between the peak from the first spectrum $\nu _{S1}$ and the peak from the second spectrum $\mu _{S2}$ as the classification problem, where the cluster membership of $\nu _{S1}$ is a true class and cluster membership of $\nu _{S2}$ is a predicted class. Thus, the alignment problem can be treated as a classification problem, and performance metrics such as accuracy, precision, and recall can be computed, as shown in Figs S13–S19. Since those metrics do not include the information about the peaks that were not matched, we define two auxiliary classes: $u_{S1}$, which includes the number of unmatched peaks from the S1 spectrum divided by the classes they belong to, and $u_{S2}$, which includes the unmatched peaks from the S2 spectrum divided by the class they belong to. Then we can extend the confusion matrix by adding a row corresponding to the $u_{S2}$ class and a column corresponding to the $u_{S1}$ class as seen in Fig. 3b.

Then, to validate the algorithm’s performance on 2D spectra and select $\delta _{\mathrm{max}}$ and $d_{\mathrm{max}}$ parameters for further analysis, we performed a grid search for the $\delta _{\mathrm{max}}$ and $d_{\mathrm{max}}$ ranging from 0.01 to 0.1 and computed the accuracy, precision, and recall metrics from the extended confusion matrices. For comparison purposes, we also computed standard performance metrics for the mentioned range of parameters.

Based on the grid search, we selected $d_{\mathrm{max}} = 0.05$ and $\delta _{\mathrm{max}} = 0.09$, computed extended metrics for 1000 sets of synthetic spectra simulated for different random seeds. Selected parameters were also used for the chain alignment of real 2D $^{1}$H-$^{15}$N HSQC spectra of GB1 protein described in the previous section.

For the 4D dataset, extended metrics were calculated across $\delta _{\mathrm{max}}$ and $d_{\mathrm{max}}$ values ranging from 0.05 to 0.15, while for the 7D dataset, the range 0.03–0.13 was used.

To account for the larger chemical shift scale of $^{15}$N dimension in 2D spectra and C1 and C2 dimensions in 4D spectra, we scaled them by the factor 0.1. In all cases, spectra were normalized before alignment.

## Results and discussion

### Comparison with existing alignment tools on the breast cancer data

To evaluate the performance of the WNetAlign algorithm, we conducted 1540 alignments, corresponding to all possible pairwise comparisons within the PXD000484 dataset. The parameters used in all alignments were max_mz_shift = 0.005 and max_rt_shift = 800. Parameter selection was performed via grid search using a representative subset of the spectra pairs. Full details of the grid search procedure are provided in Section 1.2 of the Supplementary materials.

It should be noted that (like other alignment algorithms) the performance of WNetAlign depends on the choice of max_mz_shift and max_rt_shift parameters. However, there is a wide range of settings in which it performs reasonably. For a more detailed sensitivity analysis, see Supplementary Material.

The input to the algorithm consisted of two lists of detected features in centroided mode, where each feature is represented by a single point corresponding to the entire isotopic envelope associated with a specific peptide. Feature detection was performed using the OpenMS FeatureFinderCentroided tool.

We compared the WNetAlign algorithm with two other existing alignment tools: the recently developed DeepRTAlign [35] and the widely used OpenMS alignment tool [12].

The WNetAlign algorithm demonstrated strong performance, achieving an average precision of 0.97 and an average recall of 0.96 across all alignments. In contrast, one of the most widely used alignment tools, OpenMS, performed notably worse, with a precision of 0.89 and a recall of 0.74. The average alignment time for WNetAlign was 0.6 s. OpenMS required an average of 29 s per alignment, while DeepRTAlign required substantially longer, with an average runtime above 2 h per alignment.

The aligned dataset consists of two batches: MT and TDM, with each batch containing samples processed by a different operator. When aligning spectra from different batches, OpenMS exhibited poor performance, achieving a recall of <0.3. DeepRTAlign performed substantially better than OpenMS in this setting, but it still remained below the performance of WNetAlign, especially in terms of recall (see Table 1). Although the WNetAlign algorithm also showed reduced performance in this challenging scenario, it consistently maintained precision and recall values >0.8, demonstrating its robustness to large RT shifts introduced by operator variability. The extended analysis of this behavior is provided in Section 1.4 of the Supplementary Materials.

**Table 1:** Mean performance metrics of different alignment methods

Method	Recall	Precision	Runtime
WNetAlign	0.96	0.97	0.6 s
DeepRTAlign^a^	0.90	0.95	2 h 20 min 23 s
OpenMS	0.74	0.89	29 s

^a^Due to high computation time, DeepRTAlign was evaluated only on 70 random spectra pairs from all possible pairwise comparisons. Each spectrum from the dataset was aligned at least once.

Figure 2c presents two examples of WNetAlign alignments. In each example, lines connect matched features between spectra: blue lines indicate true positive matches, while red lines represent false positive matches. The length of each line corresponds to the RT shift between matched features. Figure 2c1 illustrates an MT–MT alignment, where both spectra originate from the MT batch, whereas Fig. 2c2 depicts an alignment between an MT spectrum and a TDM spectrum. RT shifts between MT and TDM spectra are substantially larger, which likely contributes to the poor performance of OpenMS in these cross-batch alignments.

In summary, WNetAlign outperformed both the novel, deep learning-based RT alignment tool DeepRTAlign, as well as the widely used OpenMS alignment tool in terms of accuracy, robustness, and processing speed.

### Application to multidimensional NMR data

We demonstrated that the WNetAlign algorithm can be applied to align the temperature series of GB1 spectra, allowing for the computation of temperature-dependent shifts in each dimension as shown in Supplementary Fig. S7. These distances provide a quantitative basis for estimating the effect of temperature on the variance of chemical shifts.

To assess performance, we next validated the algorithm on the ground truth constructed by simulating temperature series designed to mimic the GB1 data. WNetAlign correctly matched signals from the corresponding neighborhoods, reaching maximum accuracy between 0.87 and 0.92, precision between 0.91 and 0.94, and recall between 0.93 and 0.95. Moreover, once parameter values exceeded the average distance between peaks in one spectrum and their counterparts in replicate spectra, extended metrics stabilized, plateauing for $d_{\mathrm{max}} \geq 0.05$ and $\delta _{\mathrm{max}} \geq 0.08$ (Fig. 3d). This average inter-replicate distance arises directly from the simulation design, where spectra were generated by shifting peak positions relative to one another in a controlled manner.

Analysis of the impact of the $\delta _{\mathrm{max}}$ parameter on classification results revealed that lower values reduce accuracy, precision, and recall primarily due to unmatched peaks rather than misclassifications (Figs S25–S29). This indicates that the algorithm favors conservative matching over incorrect alignments, further supporting its robustness.

The $\delta _{\mathrm{max}}$ is data specific since it represents the expected maximum distance between the shifts of signals corresponding to the same type of functional group/nuclide in the specific experiment. However, our analysis shows that the rough estimate of the expected maximum distance between signals in replicate samples should be sufficient lower bound for the sensible $\delta _{\mathrm{max}}$.

We proved that the obtained validation results were not sample-dependent by repeated validation across 1000 independently simulated temperature series datasets. Performance metrics remained highly stable across the simulations, with standard deviations of 0.029, 0.02, and 0.013 for accuracy, precision, and recall, respectively (Figs S20–S24; Table S3).

The proposed algorithm scales effectively to higher-dimensional data. For the 4D CCNOESY spectrum, accuracy reached 0.87, while precision and recall increased with parameter values, reaching 0.96 and 0.97, respectively (Fig. S31). On the 7D dataset, performance metrics increased steadily with both $\delta _{\mathrm{max}}$ and $d_{\mathrm{max}}$, plateauing near 0.1 and reaching 0.92–0.95 for precision and recall and 0.96–1.0 for accuracy (Figs S33–S49).

Together, these results highlight WNetAlign as a robust, scalable, and interpretable framework for aligning multidimensional NMR spectra. By reducing the influence of instrumental drift and environmental variability, the method improves the comparability of replicate measurements and enhances the utility of high-dimensional NMR for comparative analysis, automated quantification, and structure elucidation. Beyond structural biology, WNetAlign is particularly relevant in metabolomics, where peak shifts across samples complicate statistical comparisons and obscure biomarker discovery, and in medical diagnostics, where reliable alignment of spectra from patient samples acquired under heterogeneous conditions is essential for identifying disease-associated signatures. By enabling accurate and automated alignment across diverse experimental conditions, WNetAlign provides a foundation for broader integration of multidimensional NMR into both fundamental and translational research.

### Runtime scaling on large datasets

**Figure 4:**
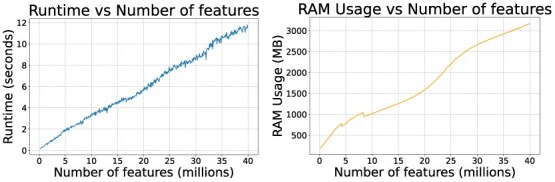
Runtime and RAM usage comparison across various input dataset sizes. The irregular behavior of RAM usage curve is due to various STL and Python data structures adjusting their overallocation strategies based on data size.

To study the algorithm’s runtime performance on large datasets, we constructed a partially artificial dataset. For each $N \in{1..500}$, we created an artificial pair of spectra from the PXD000484 dataset, repeating each spectrum $N$ times. Each repetition was shifted along the m/z axis to avoid overlap, resulting in datasets containing between 159 422 and 40 million features across the two aligned spectra. The dataset was generated so as to preserve the character of the original data, allowing us to study the effect of data size in isolation from other factors, such as varying peak density between different datasets. The results are presented in Fig. 4.

The algorithm exhibits approximately linear scaling with respect to input dataset size. In theory, the complexity is at least $n \log (n)$, although the logarithmic factor is too small to be visible in the plots. The runtime performance remains satisfactory, reaching 12 s even for the largest dataset tested.

## Conclusions

The study demonstrates that the proposed algorithm effectively aligns multidimensional spectra from LC–MS and NMR, minimizing errors caused by RT or frequency shifts. It employs a truncated Wasserstein distance and formulates alignment as a minimum-cost flow problem solved with the Network Simplex algorithm. Constraining the maximum transport distance accelerates computation, reduces false matches, and improves robustness to noise. The algorithm is suitable for comparative analyses, replicate experiments, and studies where signal shifts are intentionally introduced, such as variable-temperature NMR. Its scalability makes it applicable to large proteomics, metabolomics, and structural biology projects. Combining LC–MS and NMR with precise spectral alignment enhances the reliability of compound identification, quantification, and structural analysis.

Last but not least, the proposed algorithm can be utilized not only as an efficient method for aligning multidimensional spectra but also for efficiently estimating the high-dimensional Wasserstein distance, which has potential applications in areas such as generative modeling and flow matching techniques.

**Key Points**

- WNetAlign introduces a fast and robust spectra alignment framework based on truncated Wasserstein distance formulated as a minimum-cost flow problem.
- By constraining transport distance and using the Network Simplex algorithm, the method improves alignment accuracy while substantially reducing computational cost.
- WNetAlign outperforms widely used tools on benchmark LC–MS proteomics data and remains robust to large retention time shifts.
- The same framework naturally extends to multidimensional NMR data (2D–7D), enabling reliable alignment and peak tracking in high-dimensional bioinformatics applications.

## Supplementary Material

WNetAlign_suppl_bbag247

## Data Availability

Most of the article is based on reanalysis of publicly available datasets, see text for details. The novel NMR dataset has been uploaded to https://doi.org/10.5281/zenodo.17226285.
