# Supplementary material for: WNetAlign: fast and accurate spectra alignment using truncated Wasserstein distance and network simplex
Source: Brief Bioinform. 2026 May 25;27(3):bbag247. doi: 10.1093/bib/bbag247 (PMC13200549; doi:10.1093/bib/bbag247)
Supplement: WNetAlign_suppl_bbag247 [file wnetalign_suppl_bbag247.pdf]

Supplementary material: WNetAlign: Fast and Accurate Spectra Alignment Using Truncated Wasserstein Distance and Network Simplex

Justyna Król, Maria Bochenek, Sylwia Jopa, Krzysztof Kazimierczuk, Anna Gambin, Michał Startek

March 12, 2026

1 LC-MS data

1.1 Ground truth for PXD000484 dataset alignment

To construct a ground truth reference for feature alignment in the PXD000484 dataset, we analyzed the raw mass spectrometry files using MaxQuant version 2.6.7.0. Protein sequence information was sourced from the human proteome FASTA file (accession UP000005640) obtained from the UniProt repository.

Peptide-spectrum matches (PSMs) were filtered to retain only high-confidence identifications, defined as those with a posterior error probability (PEP) of < 0.01. Additionally, identifications flagged as reverse hits or potential contaminants were excluded to ensure reliability.

The resulting high-confidence peptide identifications were used to annotate the features detected by OpenMS. For each pairwise alignment of spectra, the ground truth was defined as the set of OpenMS feature pairs that were annotated with the same high-confidence peptide sequence. This provided a peptide-level reference standard for evaluating the accuracy of alignment.

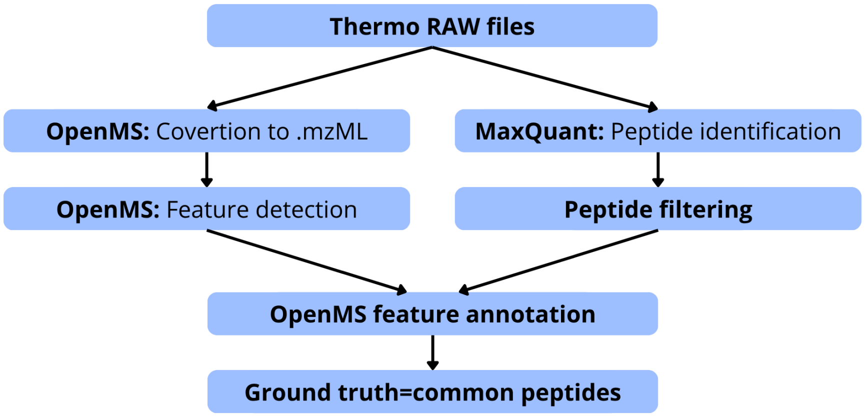

Supplementary Figure S1: Pipeline for establishing ground truth for PXD000484 dataset alignment.

1.2 Parameter gridsearch for PXD000484 dataset

The parameter grid search was conducted using a subset of the available spectra. Specifically, 50% of spectra were randomly selected. Each spectrum within this subset was then aligned to two other randomly chosen spectra from the same subset. Alignments were performed across a grid of parameter combinations that varied both the maximum retention time (RT) shift and the maximum m/z shift. For each alignment, recall and precision were calculated as described in the Results section. The outcomes of this process are summarized in Figure S2.

The optimal parameters selected for final alignments were:

Maximum retention time (RT) shift: 800

Maximum m/z shift: 0.004

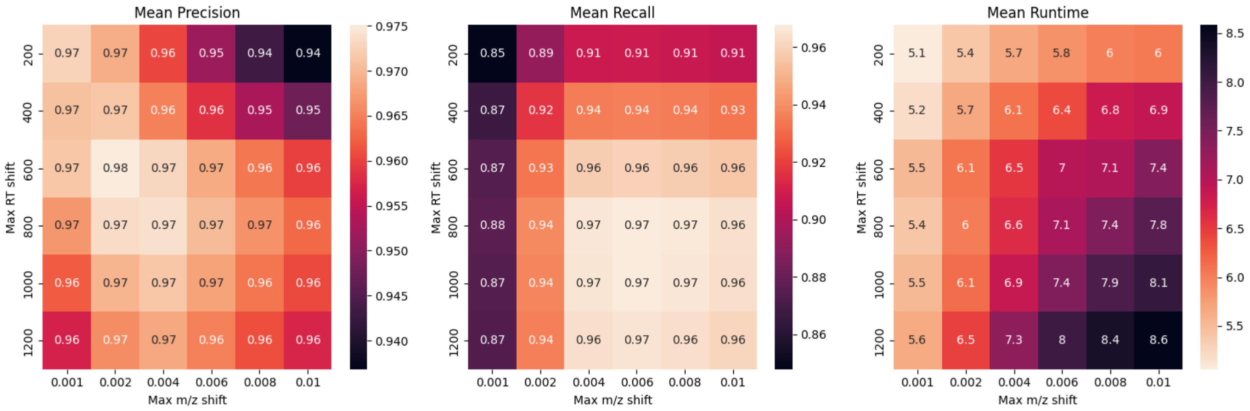

Supplementary Figure S2: Results of the parameter gridsearch on a fraction of PXD000484 alignments.

1.3 Comparison on LC-MS benchmark dataset

The benchmark dataset selected to test the WNetAlign algorithm alongside other established algorithms is the P1 dataset, previously used in [3] to evaluate various LC-MS alignment methods. Escherichia coli protein extracts from cells in the exponential growth phase were diluted in digestion buffer, denatured, and digested with trypsin. The mixtures were then analyzed using automated LC-LC-MS/MS.

The dataset consists of two runs with different injection volumes, referred to as the P1_1 and P1_2 datasets. Each run includes six spectra corresponding to different fractions (labeled 0, 20, 40, 60, 80, and 100). In this analysis, we will align spectra from the same fraction between the P1_1 and P1_2 runs.

The data was available in the .featureXML format, meaning that it had been previously analyzed by a feature-finding program.

The ground truth was established manually by the Lange et. al (2008) [3], with the use of detailed data unavailable to the alignment procedures. It consists exclusively of features that can be annotated with a reliable peptide identification. As a result, the proportion of features with a corresponding match in the other spectrum is very low. On average, only 5.8% of features in each spectrum are represented in the ground truth.

The dataset was used to compare WNetAlign with Alignstein [5], an alternative alignment algorithm based on optimal transport theory. The algorithms were evaluated under two conditions: (i) aligning spectra filtered to include only features present in the ground truth, as described in [5], and (ii) aligning the complete, unfiltered spectra.

Algorithm	Mean Precision	Mean Recall	F-score	Time (s)
Alignstein	1.000	0.951	0.975	0.516
WNetAlign	0.999	0.965	0.982	0.005

Supplementary Table S1: Alignment performance (filtered data).

In the first scenario, where filtered spectra were used, both algorithms achieved near-perfect alignment performance. The filtered spectra were very small, containing, on average, approximately 80 features per spectrum, which resulted in very short runtimes for both methods. Nevertheless, WNetAlign was approximately 100 times faster than Alignstein.

Algorithm	Mean Precision	Mean Recall	F-score	Time (s)
Alignstein	0.691	0.791	0.738	27.241
WNetAlign	0.838	0.868	0.853	0.075

Supplementary Table S2: Alignment performance (unfiltered data).

In the second scenario, WNetAlign outperformed Alignstein across all evaluated metrics. The average size of the unfiltered spectra was approximately 1,800 features per spectrum, which remains substantially smaller than typical real-world datasets (e.g., 37,000 features per spectrum in the PXD000484 dataset). On average, WNetAlign completed these alignments in 0.07 s, whereas Alignstein required 27.2 s per alignment.

It is important to note that, according to the original Alignstein paper [5], the algorithm outperformed all other established methods for LC–MS feature alignment, making it state-of-the-art. However, the results reported in that study differ from those obtained in the present work, possibly due to an error in the evaluation script previously used in [3] and [5]. In this study, both Alignstein and WNetAlign were evaluated using a corrected procedure to ensure consistency. In light of the discrepancy with the original results, we chose not to include direct comparisons with other established algorithms that had been evaluated using the possibly flawed script.

1.4 Impact of retention time shifts magnitude on alignment performance

To evaluate the robustness of the alignment method to large retention time (RT) shifts, we simulated increasing RT deviations between two spectra. Starting from an original spectrum S (that was the 100825O2c1_MT-AU-0044-2010-08-15_038 spectrum from the PXD000484 dataset, containing 39764 features), a set of shifted spectra S_{shift} was generated by perturbing both m/z and RT values. The m/z values were modified by adding Gaussian noise (sampled independently for each feature) scaled to remain within the maximum allowed mass tolerance (max_mz_shift = 0.005). RT values were shifted similarly, by adding a systematic offset with small Gaussian noise proportional to the shift magnitude. The RT offset was varied from 0 to 1400 seconds in steps of 100 seconds. Alignment performance was evaluated using precision and recall, where a match was considered correct if the aligned features shared the same feature identifier.

We first evaluated performance using a fixed tolerance of `max_rt_shift = 1200` seconds while increasing the simulated RT shift. As shown in Figure S3, precision and recall remain high for small shifts and decrease only when the true RT shift approaches or exceeds the allowed tolerance.

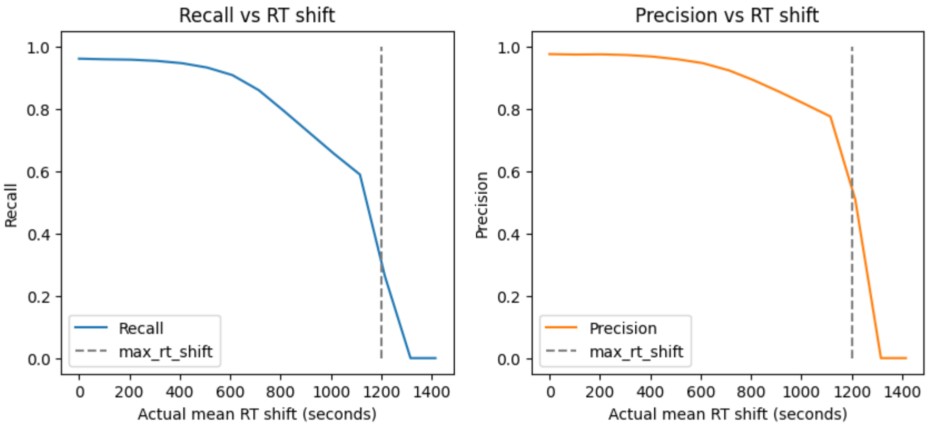

Supplementary Figure S3: Precision and recall as a function of simulated RT shift using a fixed tolerance of `max_rt_shift = 1200` seconds.

Next, for each simulated S_{shift} we evaluated alignment across a range of RT tolerances (`max_rt_shift` from 100 to 1400 seconds). The resulting precision and recall heatmaps are shown in Figure S4. Performance primarily depends on whether the allowed tolerance exceeds the true RT shift. When `max_rt_shift` is smaller than the shift, both metrics decrease because correct matches fall outside the RT window. Once the tolerance exceeds the true shift, precision and recall quickly approach high values and remain stable even for larger tolerances.

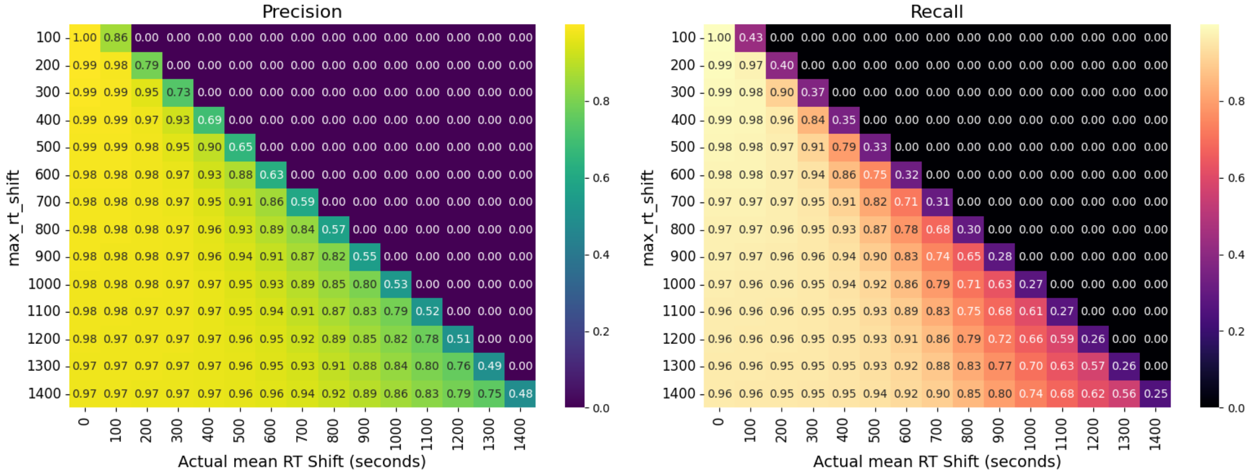

Supplementary Figure S4: Precision (left) and recall (right) as a function of simulated RT shift and the RT tolerance used during alignment.

These results indicate that `max_rt_shift` can be set conservatively (i.e., relatively large) without degrading precision, allowing the parameter to be chosen greedily while maintaining high alignment accuracy.

This analysis explains the behavior of WNetAlign algorithm on the PXD000484 dataset, which contains two batches (MT and TDM) processed by different operators. RT shifts between batches are substantially larger than those observed within a batch. Box plots comparing cross-batch

alignment performance are shown in Figure S5. For all methods, only the 70 pairs successfully aligned by DeepRTAlign were included.

WNetAlign maintains high precision and recall despite the large cross-batch RT shifts. This robustness stems from its alignment strategy based on the truncated Wasserstein distance, which matches feature distributions while tolerating large RT differences. Importantly, setting `max_rt_shift` to a large value (800 s) did not reduce performance for within-batch alignments while enabling robust cross-batch alignment.

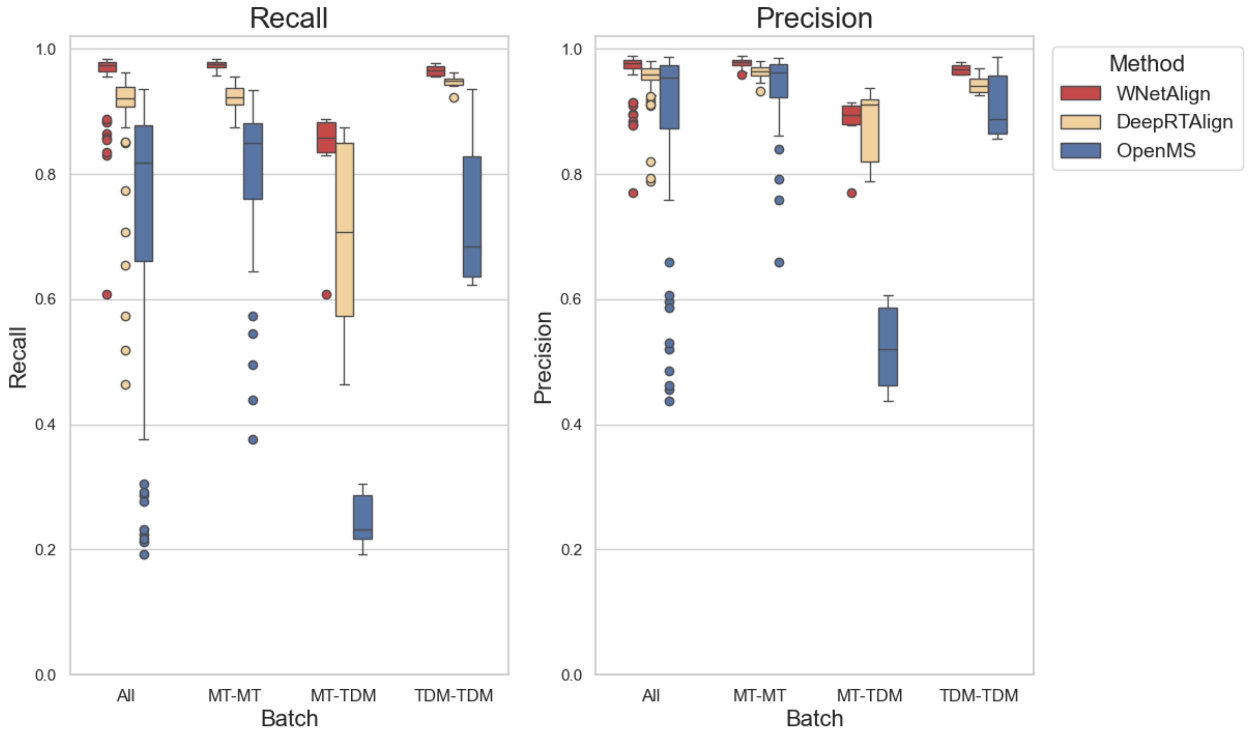

Supplementary Figure S5: Cross-batch alignment performance on the PXD000484 dataset for three alignment methods. Only the 70 pairs successfully aligned by DeepRTAlign are shown.

2 2D NMR spectra

2.1 ^{1}H-^{15}N HSQC NMR spectra

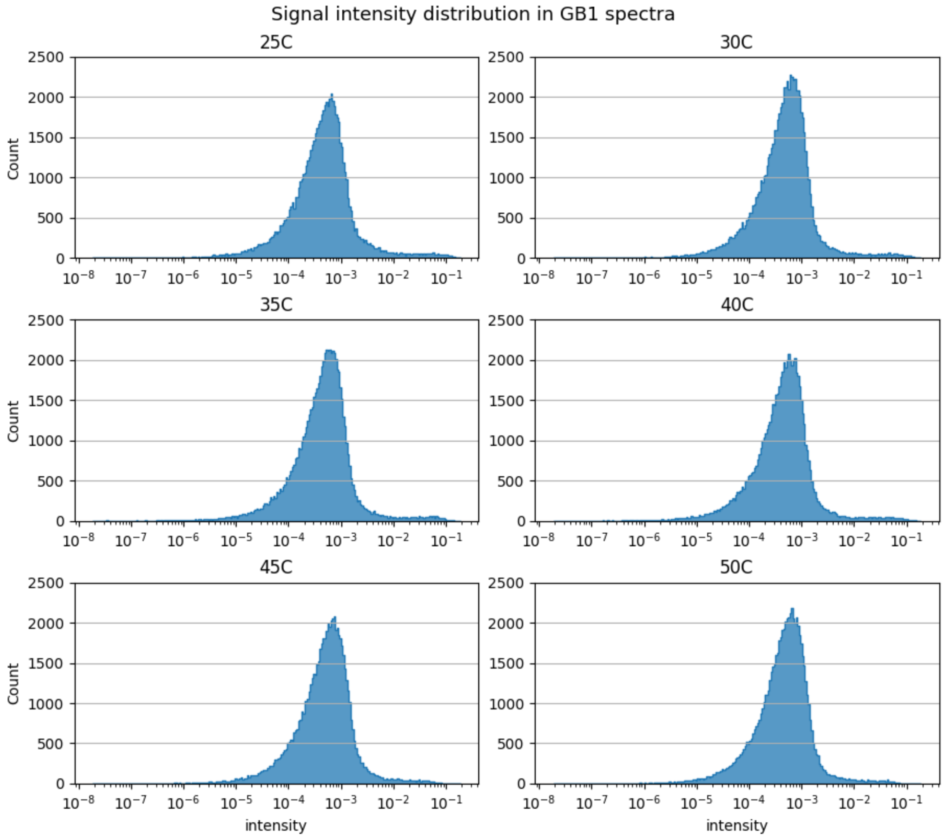

Supplementary Figure S6: Data distribution of 2D ^{1}H-^{15}N HSQC spectra of GB1 protein. The horizontal axis shows the decimal logarithm of peak intensities.

2.1.1 Chain alignment of ^{1}H-^{15}N HSQC NMR spectra

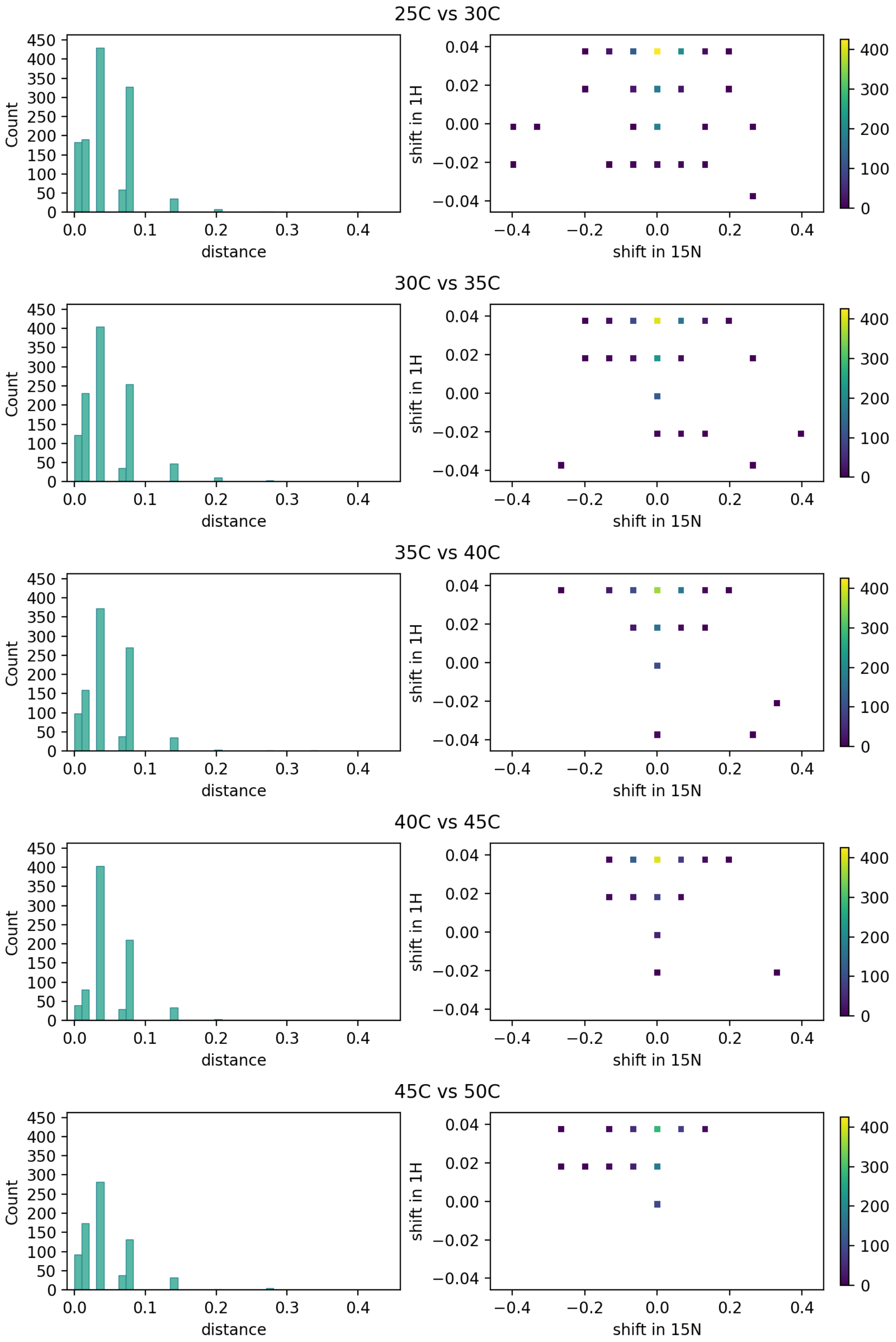

Supplementary Figure S7: Histograms of the distance (left column), shift in ^{15}N vs ^{1}H dimensions (right column) between matched points in aligned 2D ^{1}H-^{15}N HSQC spectra of GB1 protein.

2.2 Simulated ^{1}H-^{15}N HSQC NMR spectra

2.2.1 Simulating ^{1}H-^{15}N HSQC NMR temperature series for validation

To establish ground truth for validating the alignment algorithm, we generated a synthetic series of spectra designed to mimic the original 2D ^{1}H-^{15}N HSQC spectra of GB1 protein.

We first clustered the reference spectrum acquired at $25°C$ using the K-means algorithm with $k = 54$. The number of clusters was selected based on visual inspection of the spectrum and supported by hierarchical clustering of the same dataset. The clusters defined distinct classes of peaks that were then independently shifted to create the series of six spectra, where the first spectrum ($25°C$) was the original 2D ^{1}H-^{15}N HSQC spectrum measured at 25°C, and the latter five were synthetic spectra ($30°C$, $35°C$, $40°C$, $45°C$, $50°C$) created by shifting the peaks from the preceding spectrum in a series.

To generate peaks $\mathbf{p}'_i = \begin{pmatrix} x'_{1\mathrm{H}} & x'_{15\mathrm{N}} \end{pmatrix}^\intercal$ of a synthetic spectrum for temperature $t \in (30, 35, 40, 43, 50)$, the peaks from the previous spectrum in a series $\mathbf{p}_i = \begin{pmatrix} x_{1\mathrm{H}} & x_{15\mathrm{N}} \end{pmatrix}^\intercal$, were shifted according to the formula

$$\begin{aligned} \mathbf{p}'_i &= \mathbf{p}_i + \mathbf{x}_t + \mathbf{x}_c \\ &= \begin{pmatrix} x_{1\mathrm{H}} \\ x_{15\mathrm{N}} \end{pmatrix} + \begin{pmatrix} x_{1\mathrm{H},t} \\ x_{15\mathrm{N},t} \end{pmatrix} + \begin{pmatrix} x_{1\mathrm{H},c} \\ x_{15\mathrm{N},c} \end{pmatrix}, \end{aligned}$$

where $\mathbf{x}_t$ is the 2-dimensional vector of shifts for the temperature t, and $\mathbf{x}_c$ is the 2-dimensional vector of shifts for the class of peaks c that $\mathbf{p}_i$ belongs to. For each $t \in (30, 35, 40, 43, 50)$ the temperature-dependent shifts were drawn uniformly from the interval $[0, 0.01]$ for the ^{1}H dimension and $[0, 0.005]$ for the ^{15}N dimension. Cluster-dependent shifts $\mathbf{x}_c$ were drawn from $[0.015, 0.035]$ interval for ^{1}H and $[-0.01, 0.015]$ for ^{15}N for each cluster c independently. The exact values of the shifts are provided in the GitHub repository https://github.com/michalsta/wnetalign.

To simplify the evaluation and ensure well-defined performance metrics, we did not simulate the disappearance of peaks for increasing temperature. Consequently, each spectrum in the simulated series contained the same number of peaks per cluster, thereby permitting a theoretically exact one-to-one mapping between spectra.

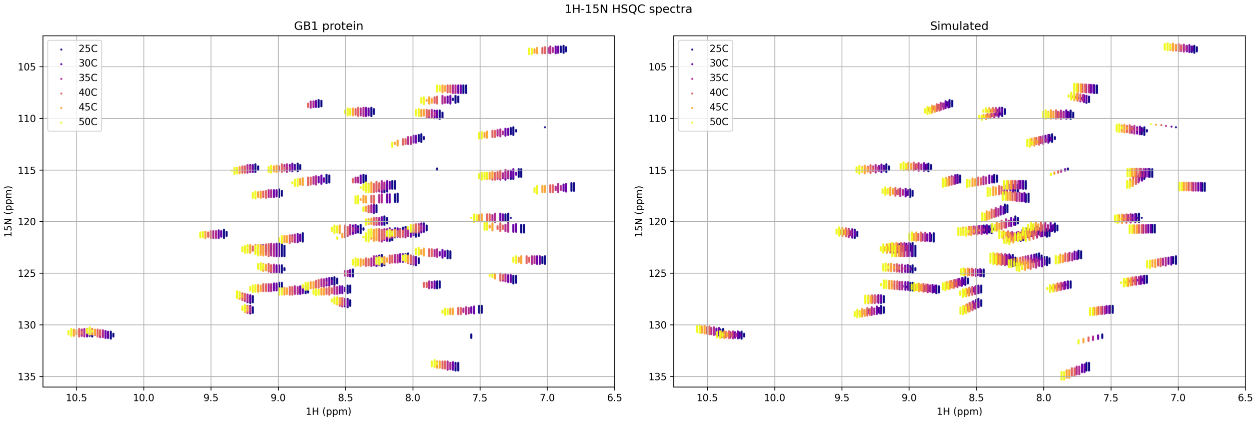

Supplementary Figure S8: Series of 2D ^{1}H-^{15}N HSQC spectra of GB1 protein measured at different temperatures (left) and simulated series of spectra mimicking the general properties of the original data (right) that was used for validation, shown in Section 2.2.2.

2.2.2 Performance metrics on simulated 2D data

Let's consider the matching between the peak from the first spectrum ν_{S1} and the peak from the second spectrum μ_{S2} as the classification problem, where the cluster membership of ν_{S1} is a true class and cluster membership of ν_{S2} is a predicted class. Thus, the alignment problem can be treated as a classification problem. Under this formulation, alignment quality can be evaluated using standard classification metrics such as accuracy, precision, and recall. These metrics were computed for the parameters $\delta_{\max}$ and $d_{\max}$ across the range 0.01–0.1 (Figures S9–S13).

Note that the accuracy increases when the parameters rise from 0.01 to 0.02 and drops when the parameters rise from 0.02 to 0.03 (See Figures S9-S13). The reason for this is that the average distance between peaks from one spectrum and peaks from another spectrum from the synthetic temperature series is approximately equal 0.03 due to the simulation procedure. For parameter values smaller than this threshold, fewer peaks are aligned; however, the matches are more likely to be correct, resulting in higher accuracy. Conversely, when the parameter values exceed the average distance, more incorrect matches are introduced, reducing accuracy.

To mitigate this behaviour and include the information about the peaks that were not matched, we define two auxiliary classes: u_{S1}, which includes the number of unmatched peaks from the S1 spectrum divided by the classes they belong to, and u_{S2}, which includes the unmatched peaks from the S2 spectrum divided by the class they belong to. Then we can extend the confusion matrix by adding a row corresponding to the u_{S2} class and a column corresponding to the u_{S1} class, as seen in the Figure S14.

Extended performance metrics based on this formulation were computed for $\delta_{\max}$ and $d_{\max}$ in the same parameter range $[0.01, 0.1]$ (Figures S15–S19). Unlike the original formulation, the extended metrics exhibit more stable behavior: accuracy, precision, and recall increase steadily with parameter values and plateau for $d_{\max} \geq 0.05$ and $\delta_{\max} \geq 0.08$. This demonstrates the robustness of the method.

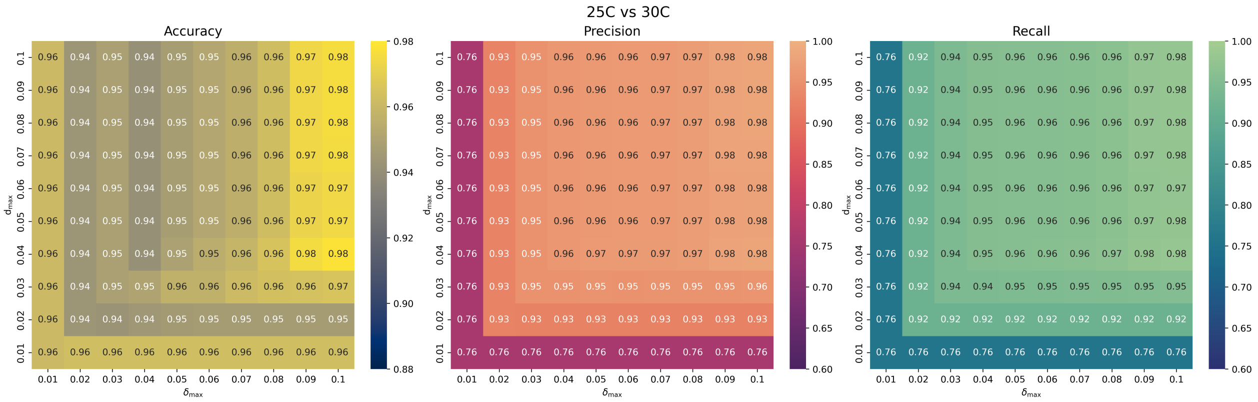

Supplementary Figure S9: Performance metrics computed for the alignment of simulated $25°C$ and $30°C$ GB1 spectra.

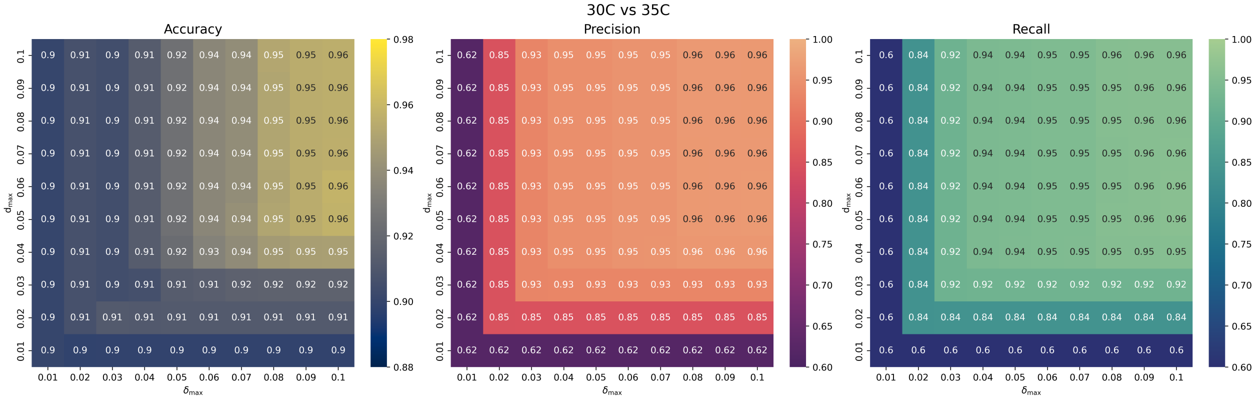

Supplementary Figure S10: Performance metrics computed for the alignment of simulated 30°C and 35°C GB1 spectra.

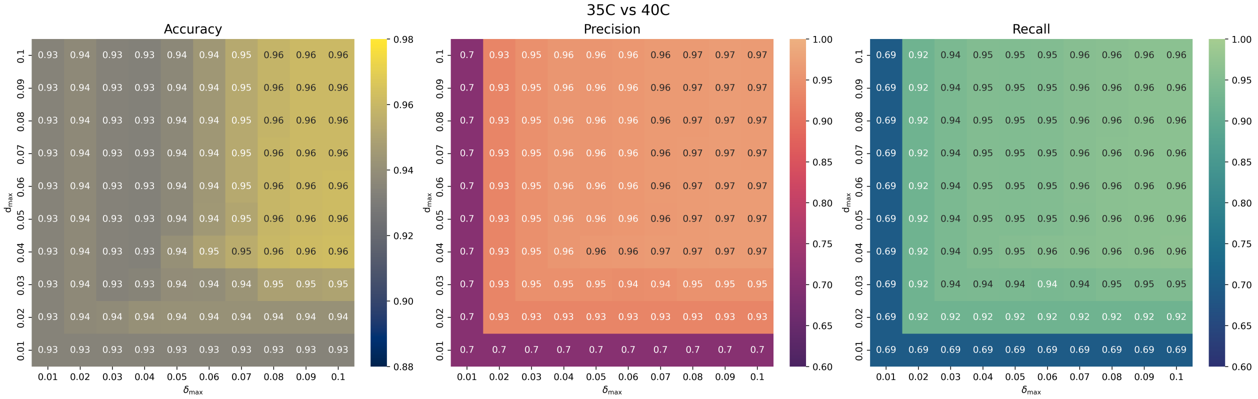

Supplementary Figure S11: Performance metrics computed for the alignment of simulated 35°C and 40°C GB1 spectra.

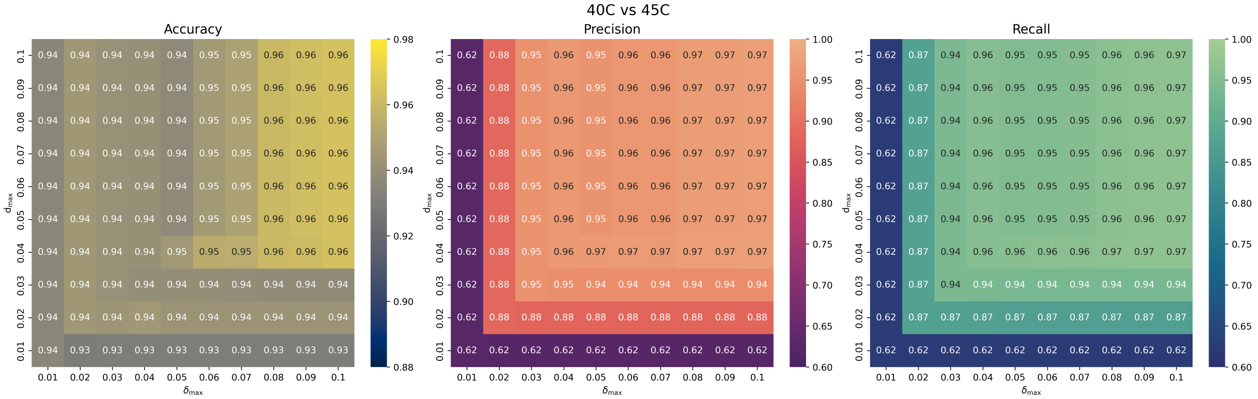

Supplementary Figure S12: Performance metrics computed for the alignment of simulated 40°C and 45°C GB1 spectra.

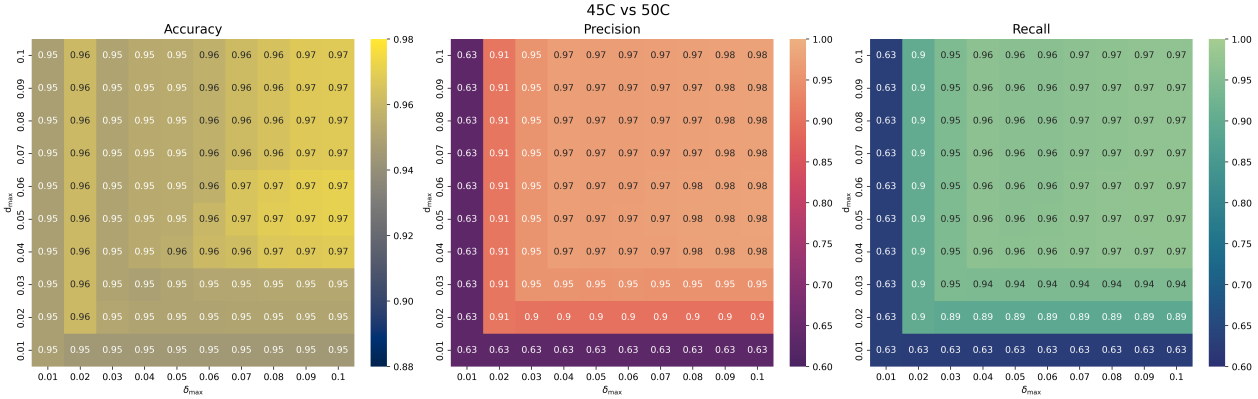

Supplementary Figure S13: Performance metrics computed for the alignment of simulated 45°C and 50°C GB1 spectra.

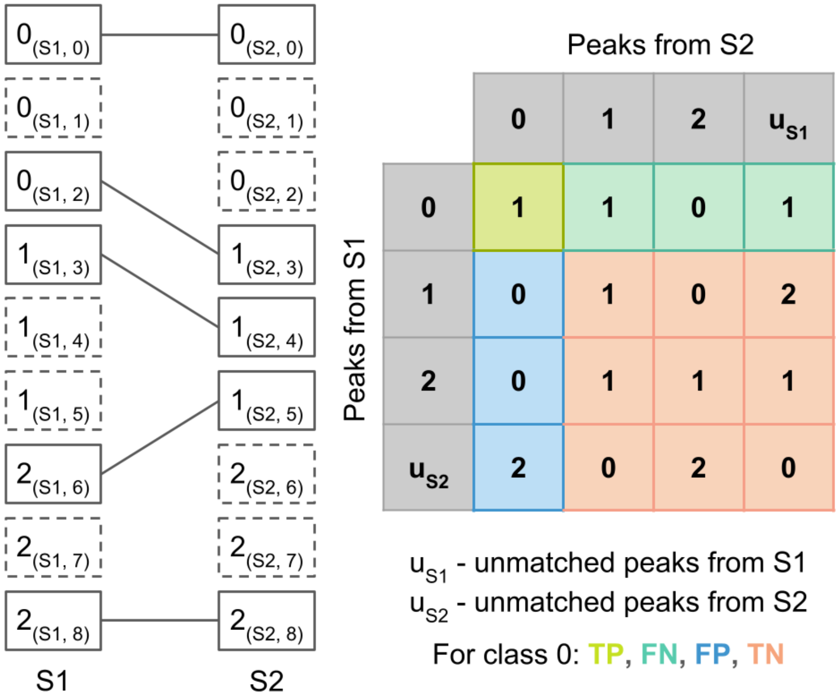

Supplementary Figure S14: Concept of the extended performance metrics. The confusion matrix is coloured by type of classification for class 0.

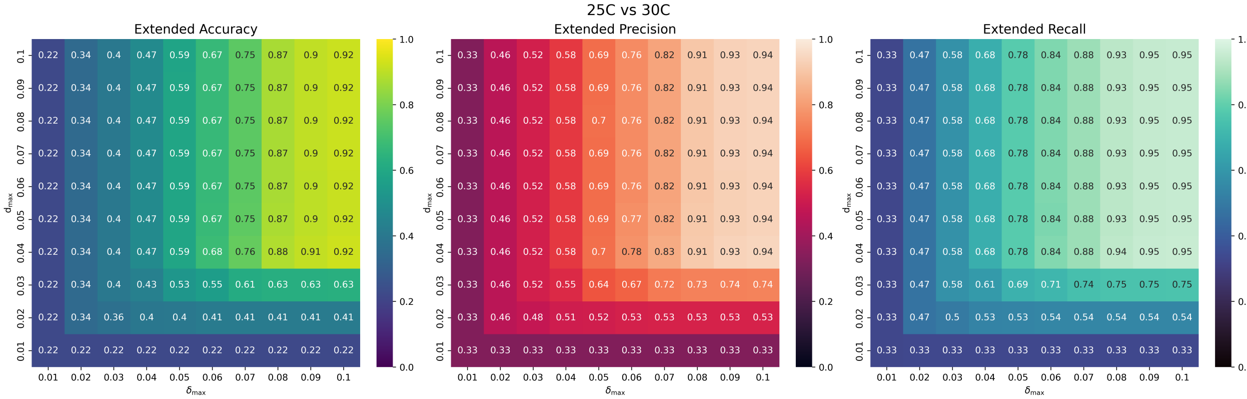

Supplementary Figure S15: Extended performance metrics computed for the alignment of simulated $25°C$ and $30°C$ GB1 spectra.

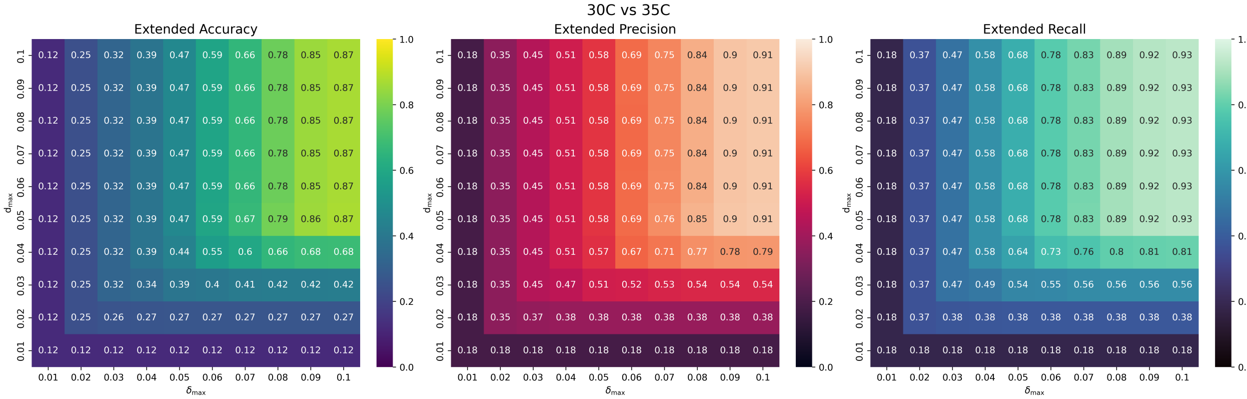

Supplementary Figure S16: Extended performance metrics computed for the alignment of simulated $30°C$ and $35°C$ GB1 spectra.

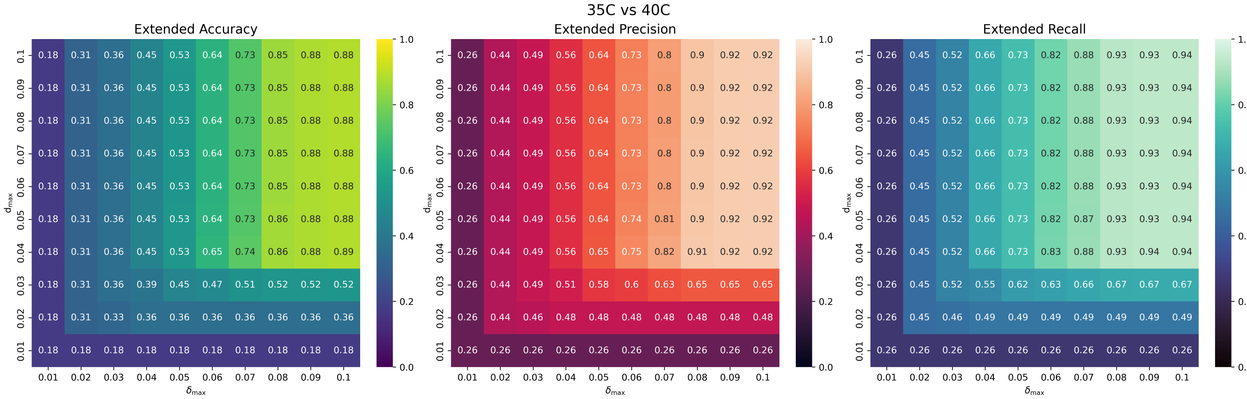

Supplementary Figure S17: Extended performance metrics computed for the alignment of simulated $35°C$ and $40°C$ GB1 spectra.

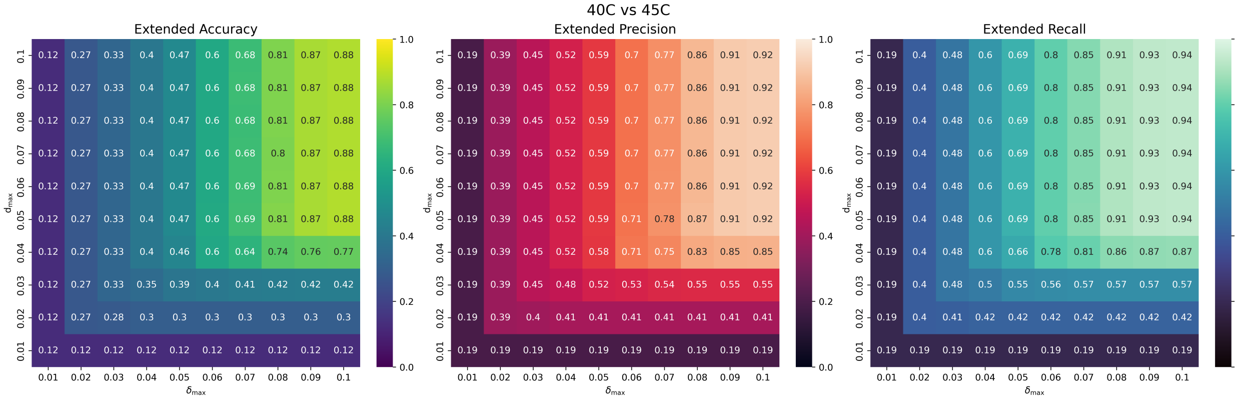

Supplementary Figure S18: Extended performance metrics computed for the alignment of simulated $40°C$ and $45°C$ GB1 spectra.

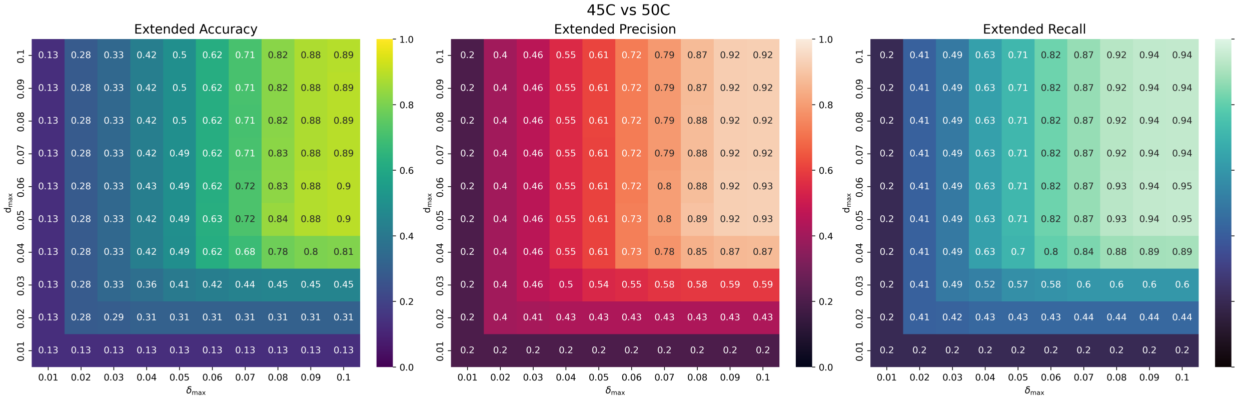

Supplementary Figure S19: Extended performance metrics computed for the alignment of simulated $45°C$ and $50°C$ GB1 spectra.

2.2.3 Robustness of the simulation procedure

To verify that performance results were not sample-dependent, the process of generating synthetic spectra was repeated 1000 times according to the procedure described in Section 2.2.1 for random seeds ranging from 0 to 999, where the spectra simulated with a random seed equal to 0 are the same as the ones used for validation described in Section 2.2.2.

We observed stable behaviour of the metrics as can be seen in Figures S20 - S24. The average values of metrics with their standard deviation are presented in Table S3.

	Accuracy	Precision	Recall
25C vs 30C	0.788 ± 0.029	0.848 ± 0.02	0.914 ± 0.013
30C vs 35C	0.787 ± 0.029	0.847 ± 0.02	0.914 ± 0.012
35C vs 40C	0.786 ± 0.029	0.847 ± 0.02	0.913 ± 0.013
40C vs 45C	0.784 ± 0.029	0.845 ± 0.02	0.911 ± 0.013
45C vs 50C	0.783 ± 0.029	0.844 ± 0.02	0.911 ± 0.013

Supplementary Table S3: Average values of extended performance metrics with the standard deviation.

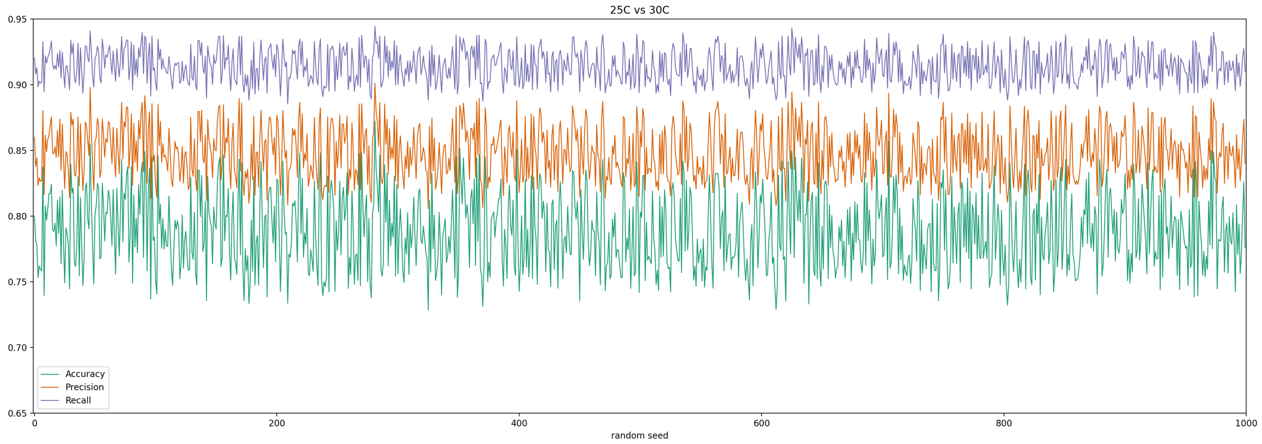

Supplementary Figure S20: Extended performance metrics computed for the alignments of 1000 pairs of 25°C and 30°C spectra simulated using different random seeds.

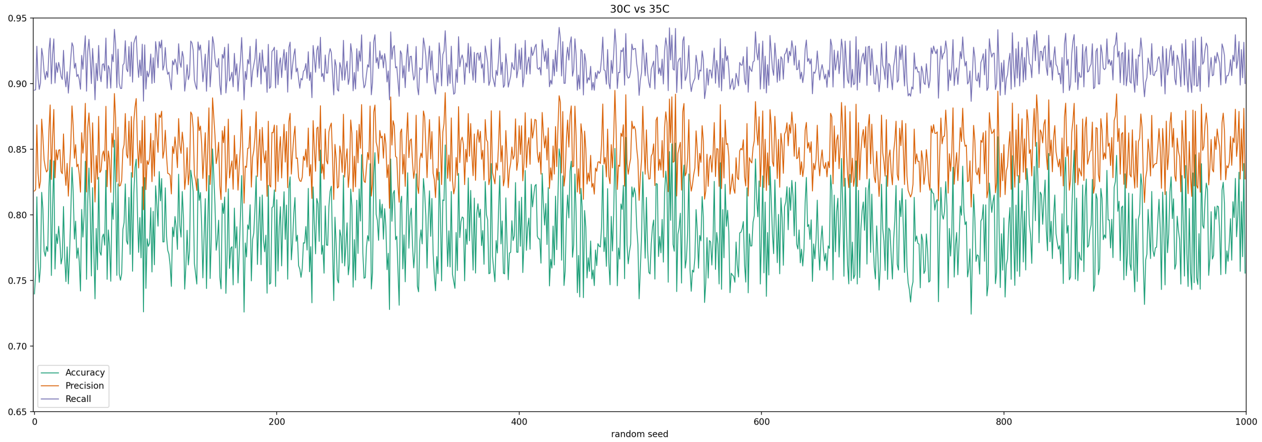

Supplementary Figure S21: Extended performance metrics computed for the alignments of 1000 pairs of 30°C and 35°C spectra simulated using different random seeds.

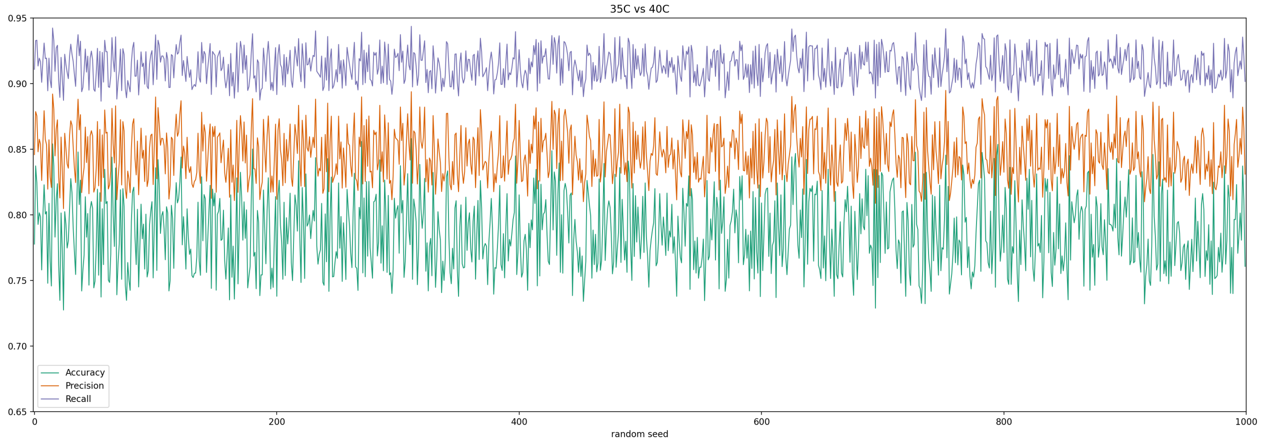

Supplementary Figure S22: Extended performance metrics computed for the alignments of 1000 pairs of 35°C and 40°C spectra simulated using different random seeds.

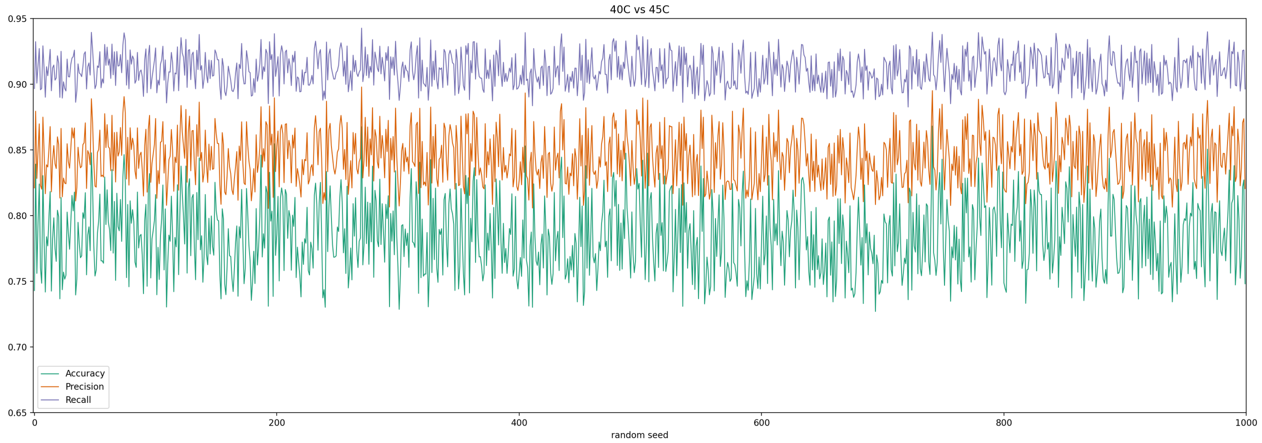

Supplementary Figure S23: Extended performance metrics computed for the alignments of 1000 pairs of 40°C and 45°C spectra simulated using different random seeds.

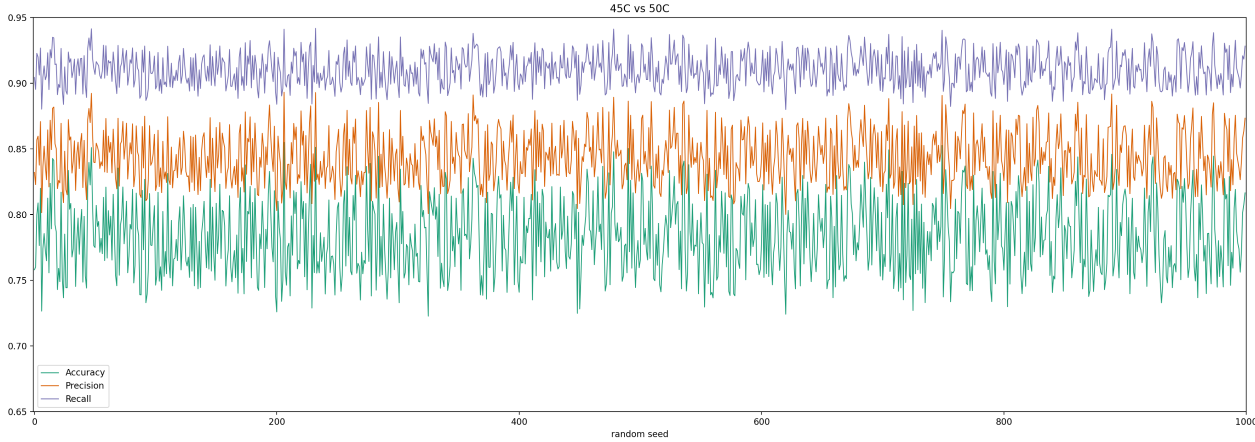

Supplementary Figure S24: Extended performance metrics computed for the alignments of 1000 pairs of 45°C and 50°C spectra simulated using different random seeds.

2.2.4 Confusion matrices

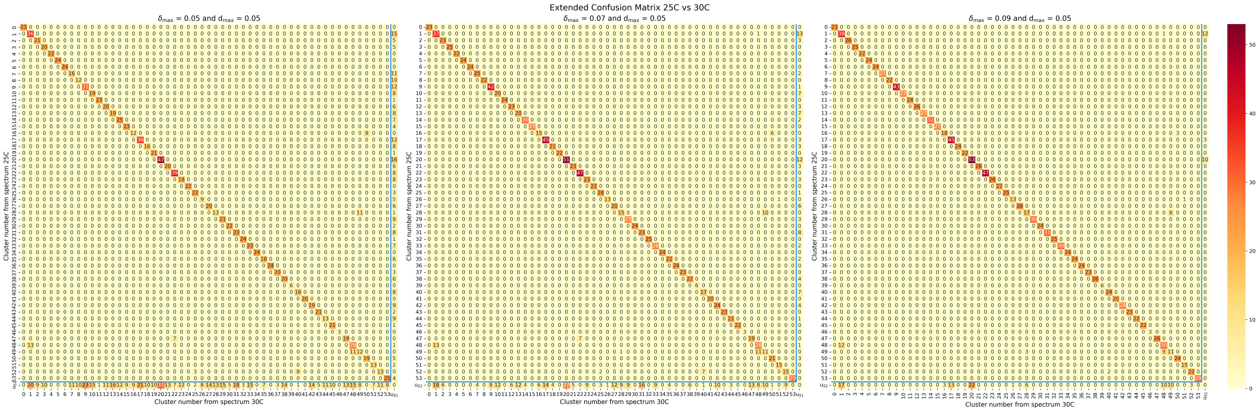

Supplementary Figure S25: Extended confusion matrices for the alignment of 25°C and 30°C simulated spectra for different $\delta_{\max}$ values.

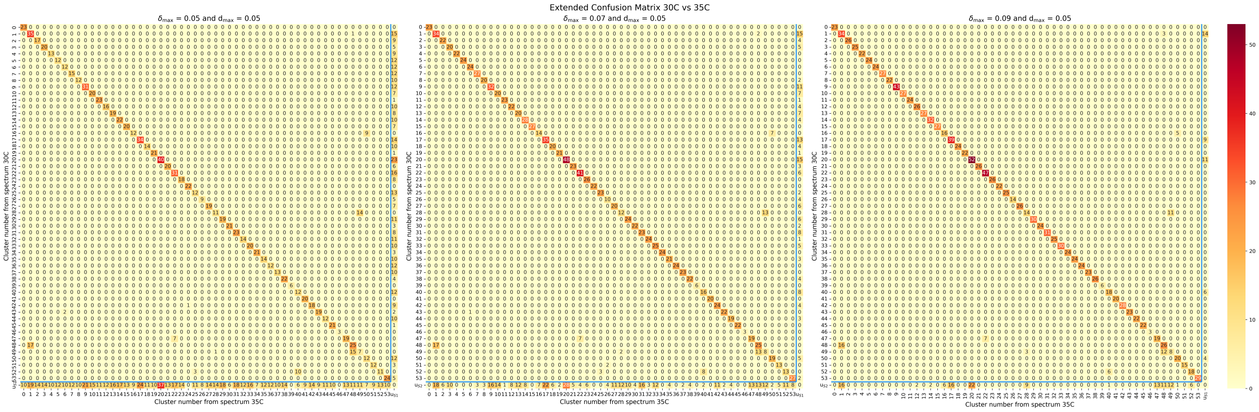

Supplementary Figure S26: Extended confusion matrices for the alignment of 30°C and 35°C simulated spectra for different $\delta_{\max}$ values.

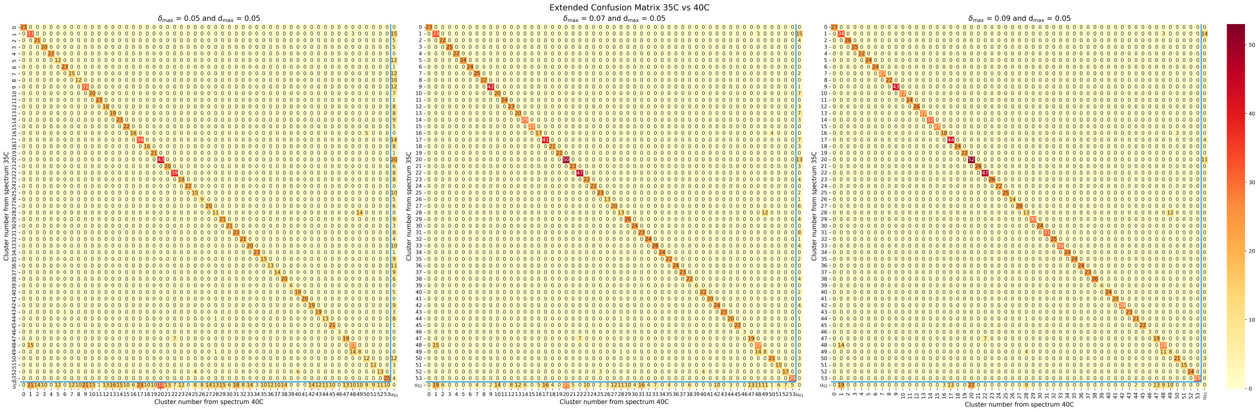

Supplementary Figure S27: Extended confusion matrices for the alignment of 35°C and 40°C simulated spectra for different $\delta_{\max}$ values.

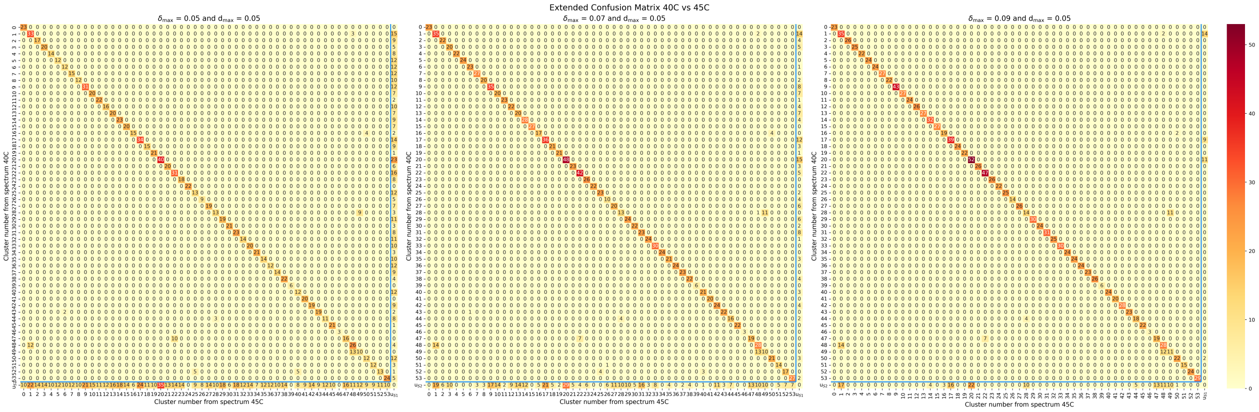

Supplementary Figure S28: Extended confusion matrices for the alignment of 40°C and 45°C simulated spectra for different $\delta_{\max}$ values.

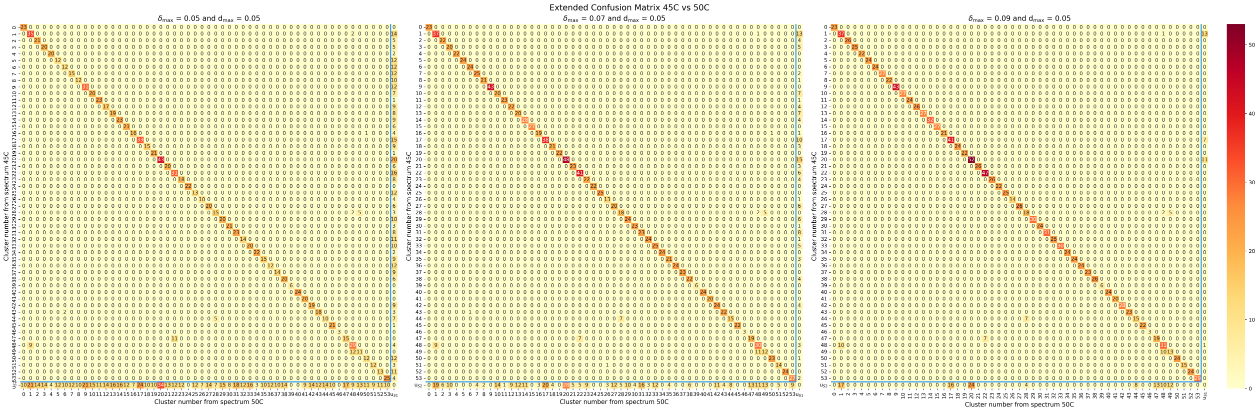

Supplementary Figure S29: Extended confusion matrices for the alignment of 45°C and 50°C simulated spectra for different $\delta_{\max}$ values.

3 4D NMR spectra

To demonstrate that the algorithm can be applied to higher-dimensional spectra, we utilized the CCNOESY (aliphatic) 4D spectrum of the 2LX7 PDB structure of the SH3 domain of growth arrest-specific protein 7 (GAS7) published as part of the 100-protein NMR spectra dataset [2]. To reduce noise, only peaks with intensities greater than 10% of the maximum were retained.

The corresponding replicate spectrum was generated by sampling shifts in each dimension for each class independently. Shifts were drawn uniformly from the $[0.025, 0.045]$ interval for C1 and C2 dimensions, and from the $[0.015, 0.025]$ interval for H1 and H2 dimensions. Class assignments were determined based on the closest local maximum (Euclidean distance) computed on the original spectrum using a multidimensional maximum filter with a window size equal to 5.

Standard and extended performance metrics were computed for the $\delta_{\max}$ and $d_{\max}$ parameters ranging from 0.05 to 0.15.

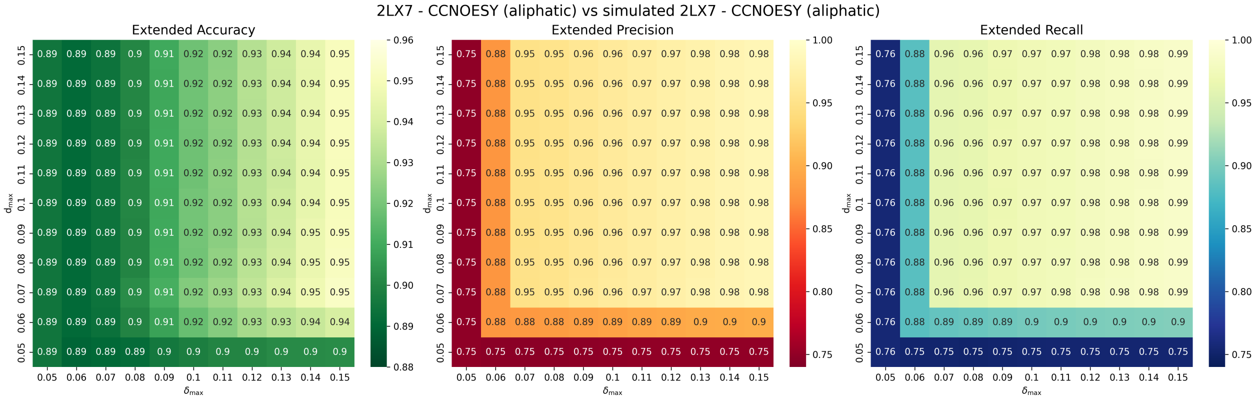

Supplementary Figure S30: Standard performance metrics computed between the 4D CCNOESY (aliphatic) NMR spectrum of 2LX7 protein and the shifted 4D CCNOESY (aliphatic) NMR spectrum of 2LX7 protein. A synthetic spectrum was obtained using the closest local maximum to determine the class of the spectrum peaks.

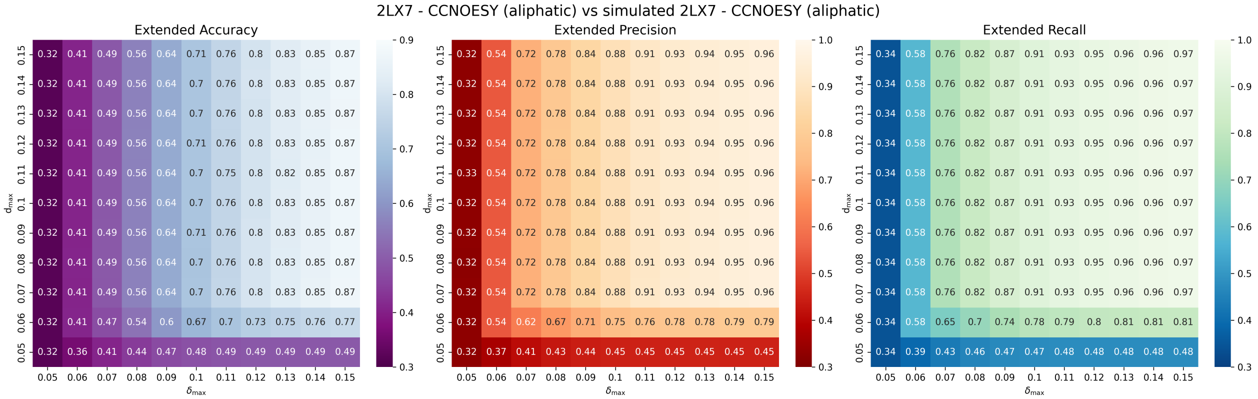

Supplementary Figure S31: Extended performance metrics computed between the 4D CCNOESY (aliphatic) NMR spectrum of 2LX7 protein and the shifted 4D CCNOESY (aliphatic) NMR spectrum of 2LX7 protein. A synthetic spectrum was obtained using the closest local maximum to determine the class of the spectrum peaks.

4 Hypothetical 7D NMR spectra

To demonstrate robustness of the method in even higher dimensions, we utilized 7D HN-N-CO-CA-CB-HA-HB NMR peak lists of 17 proteins published by Romero et al. (2022)[4]. The proteins' BMRB [1] Entry IDs are listed in Table S4. In practice, the hypothetical 7D spectra were constructed from peaks from a set of 2D-4D spectra as described in [4].

Peaks with missing chemical shifts in at least one dimension were discarded, resulting in 17 spectra (Table S4) with amino acid distribution shown in Figure S32. Since the hypothetical spectra were constructed from peak lists, they did not include signal intensities; thus, all intensities were set to 1.

BMRB Entry ID	number of peaks	number of peaks without missing chemical shifts
6436	1592	993
11526	2083	1730
15176	1693	1411
15179	2185	1912
15180	1112	901
15201	2078	1722
15225	1304	1063
15430	1187	829
15883	1337	1093
15884	1290	859
16296	1235	855
16445	669	537
17290	1831	1387
17483	1501	1164
19258	578	439
25118	1084	574
30205	1593	1351

Supplementary Table S4: Number of peaks in 7D NMR spectra of 17 proteins before and after removing peaks with missing values in at least one dimension.

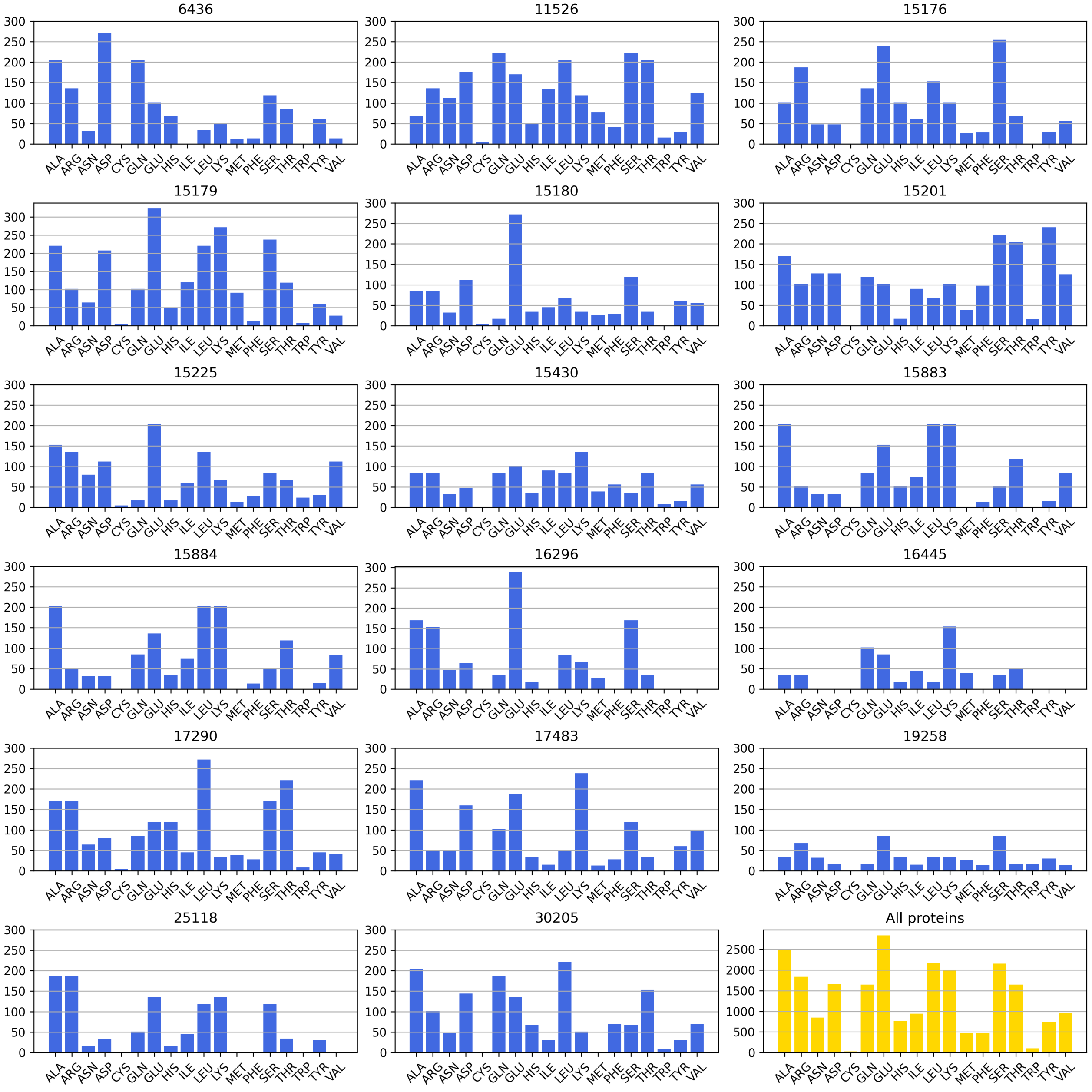

Supplementary Figure S32: Amino acids distribution in 7D HN-N-CO-CA-CB-HA-HB NMR spectra of 17 proteins.

4.1 Simulating shifted 7D NMR spectra

For each spectrum, we simulate the corresponding shifted spectrum by moving each peak depending on which amino acid class it belongs to. Let $\mathbf{p}_i$ be a 7-dimensional NMR peak, which comes from an amino acid a. We generate a peak $\mathbf{p}'_i$ by adding a vector $\mathbf{x}_i = \mathbf{x}_n + \mathbf{x}_a$ to $\mathbf{p}_i$, where $\mathbf{x}_n$ is a 7-dimensional vector of uniformly sampled baseline shifts for each nucleus, and $\mathbf{x}_a$ is a 7-dimensional

uniformly sampled vector of shifts for each nucleus for the amino acid a. Thus we have

$$\mathbf{p}_i' = \mathbf{p}_i + \mathbf{x}_i = \mathbf{p}_i + \mathbf{x}_n + \mathbf{x}_a = \mathbf{p}_i + \begin{pmatrix} x_{HN} \\ x_N \\ x_{CO} \\ x_{CA} \\ x_{CB} \\ x_{HA} \\ x_{HB} \end{pmatrix} + \begin{pmatrix} x_{HN,a} \\ x_{N,a} \\ x_{CO,a} \\ x_{CA,a} \\ x_{CB,a} \\ x_{HA,a} \\ x_{HB,a} \end{pmatrix}.$$

Values of $\mathbf{x}_n$ were uniformly sampled from the interval $(0.015, 0.025)$, while values of $\mathbf{x}_a$ for each a were independently sampled from a uniform distribution over the interval $(-0.015, 0.015)$.

4.2 Extended performance metrics of alignment of real 7D NMR spectra vs shifted 7D NMR spectra

We aligned 17 spectra pairs (original and replicate) for the $\delta_{\max}$ and $d_{\max}$ parameters ranging from 0.03 to 0.13. All spectra were normalized before the alignment. Computed extended metrics can be found in Figures S33 - S49

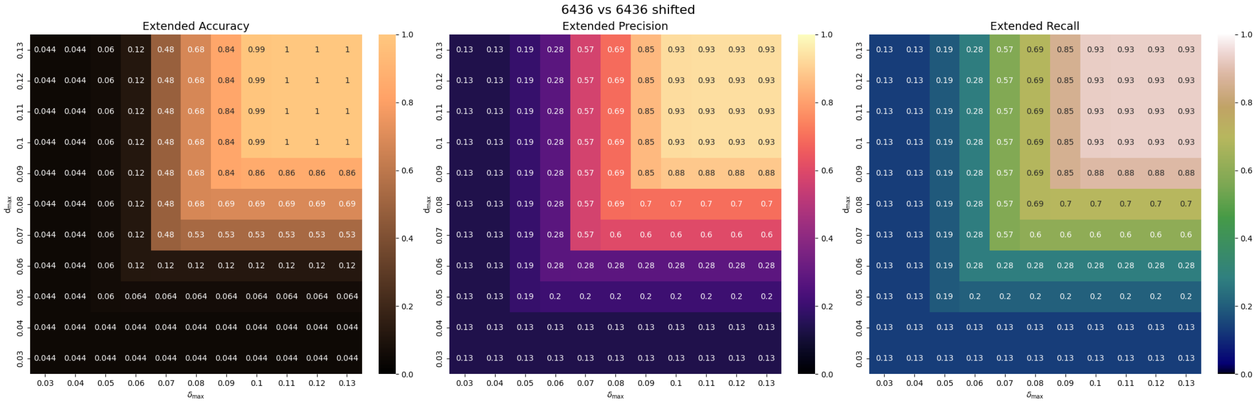

Supplementary Figure S33: Extended performance metrics computed for the alignment of the original 7D NMR spectrum of protein 6436 and the shifted 7D NMR spectrum of protein 6436.

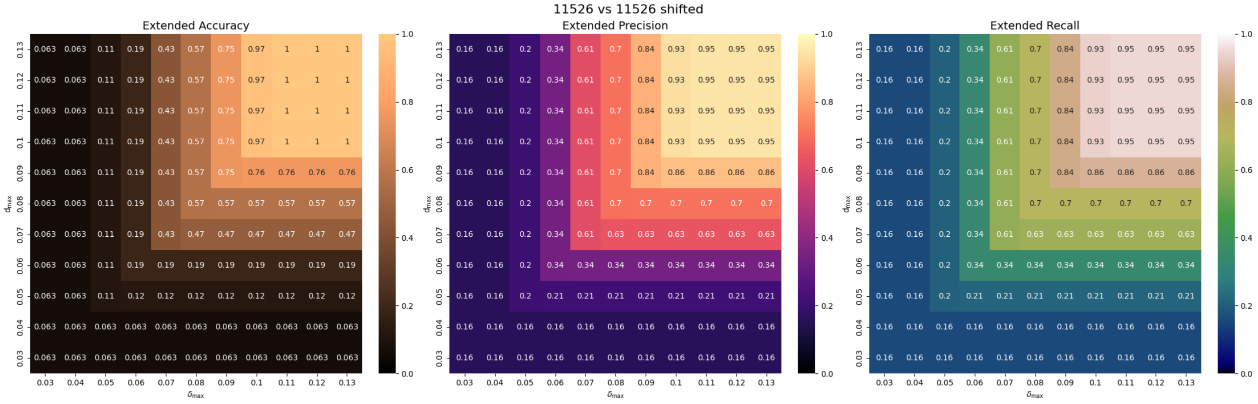

Supplementary Figure S34: Extended performance metrics computed for the alignment of the original 7D NMR spectrum of protein 11526 and the shifted 7D NMR spectrum of protein 11526.

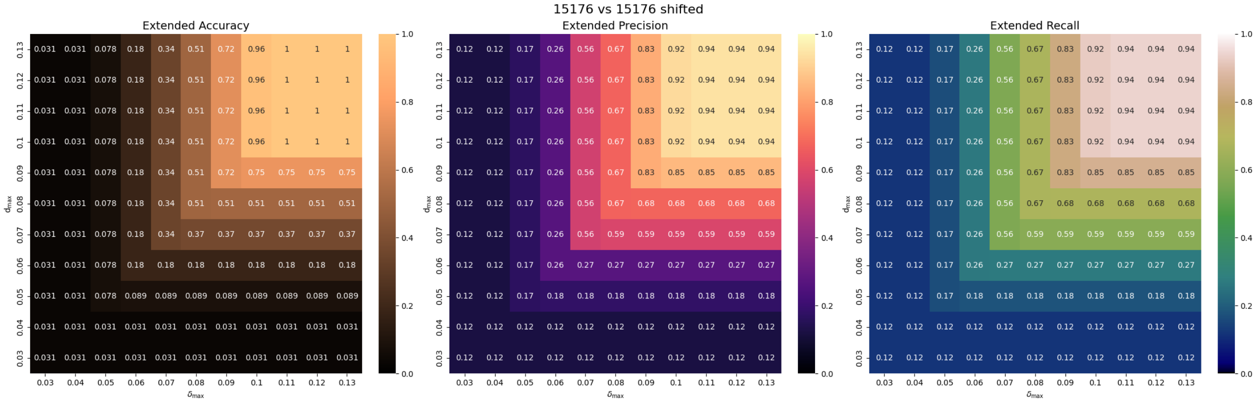

Supplementary Figure S35: Extended performance metrics computed for the alignment of the original 7D NMR spectrum of protein 15176 and the shifted 7D NMR spectrum of protein 15176.

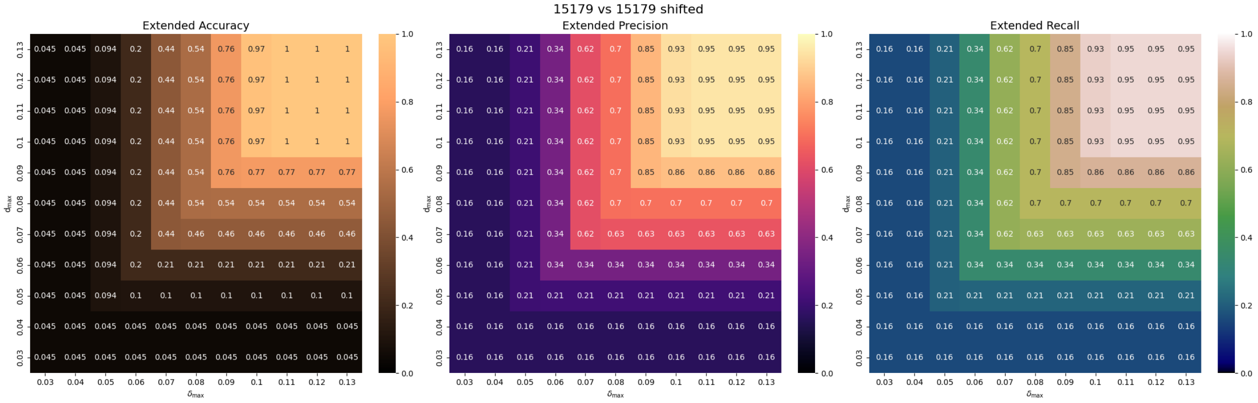

Supplementary Figure S36: Extended performance metrics computed for the alignment of the original 7D NMR spectrum of protein 15179 and the shifted 7D NMR spectrum of protein 15179.

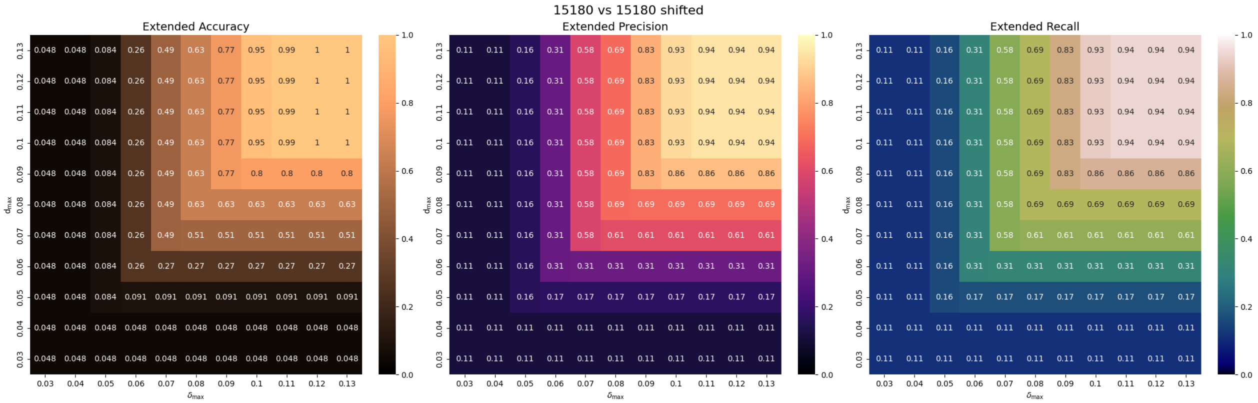

Supplementary Figure S37: Extended performance metrics computed for the alignment of the original 7D NMR spectrum of protein 15180 and the shifted 7D NMR spectrum of protein 15180.

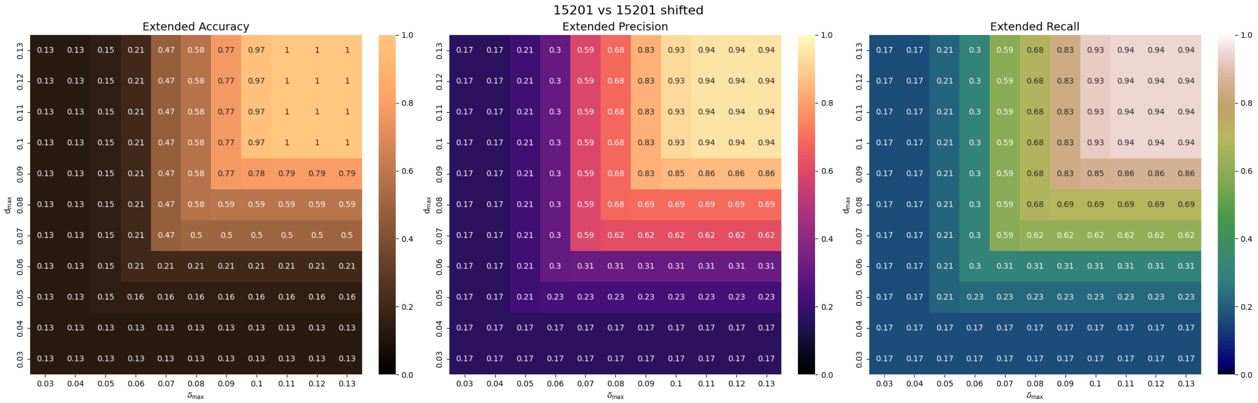

Supplementary Figure S38: Extended performance metrics computed for the alignment of the original 7D NMR spectrum of protein 15201 and the shifted 7D NMR spectrum of protein 15201.

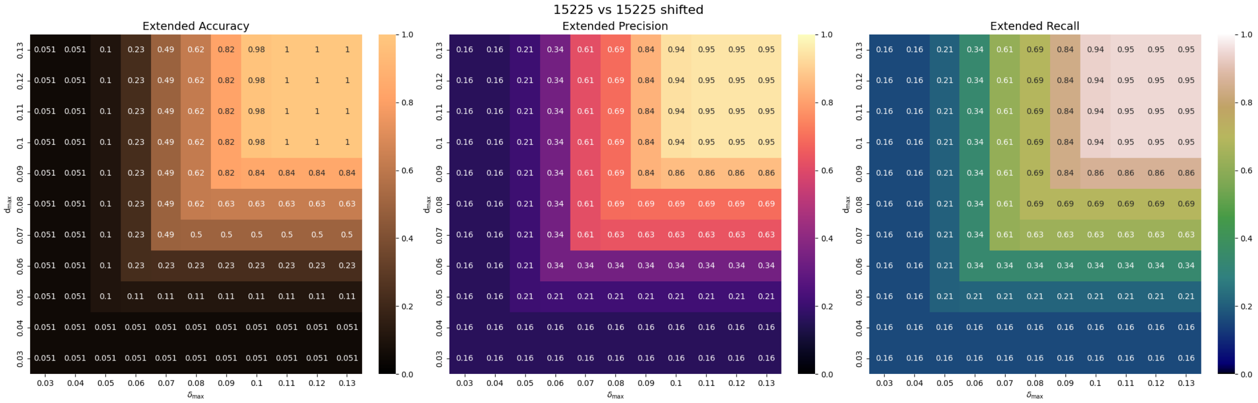

Supplementary Figure S39: Extended performance metrics computed for the alignment of the original 7D NMR spectrum of protein 15225 and the shifted 7D NMR spectrum of protein 15225.

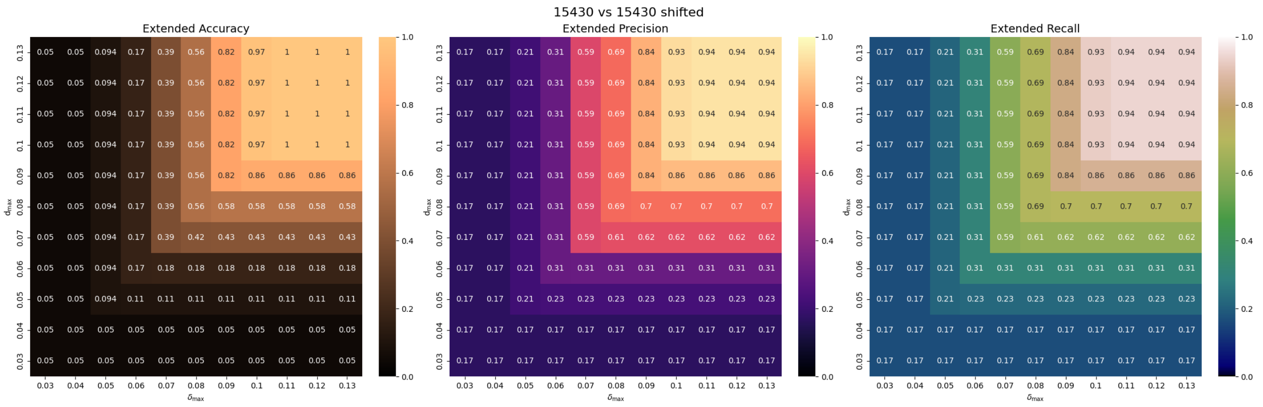

Supplementary Figure S40: Extended performance metrics computed for the alignment of the original 7D NMR spectrum of protein 15430 and the shifted 7D NMR spectrum of protein 15430.

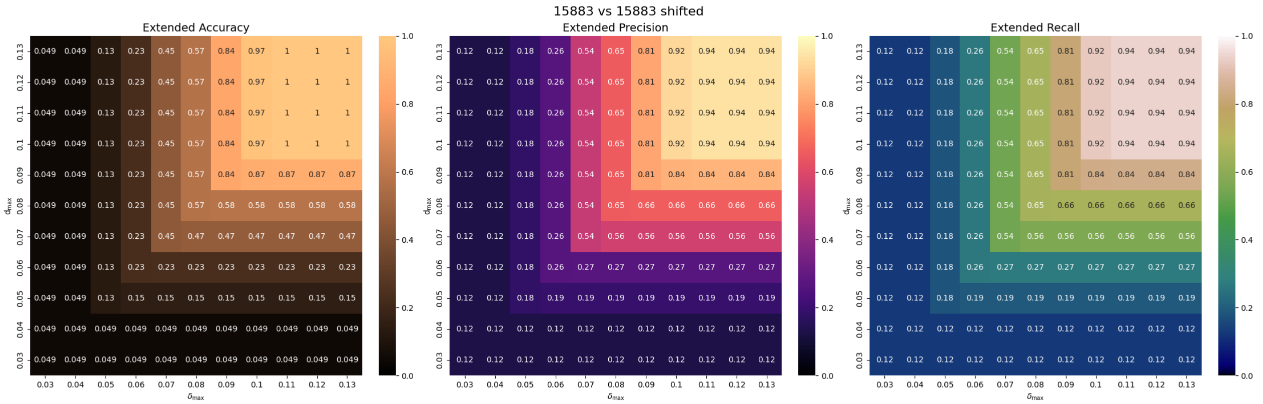

Supplementary Figure S41: Extended performance metrics computed for the alignment of the original 7D NMR spectrum of protein 15883 and the shifted 7D NMR spectrum of protein 15883.

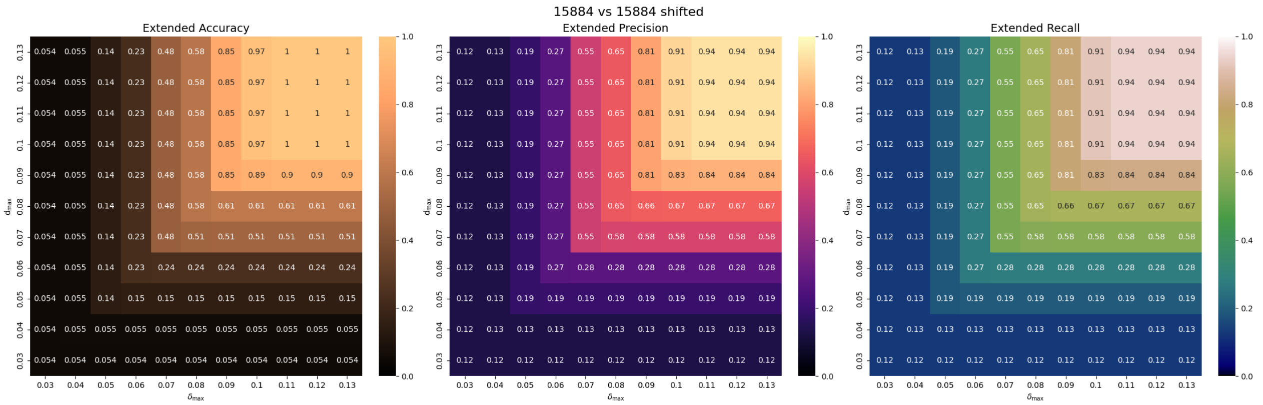

Supplementary Figure S42: Extended performance metrics computed for the alignment of the original 7D NMR spectrum of protein 15884 and the shifted 7D NMR spectrum of protein 15884.

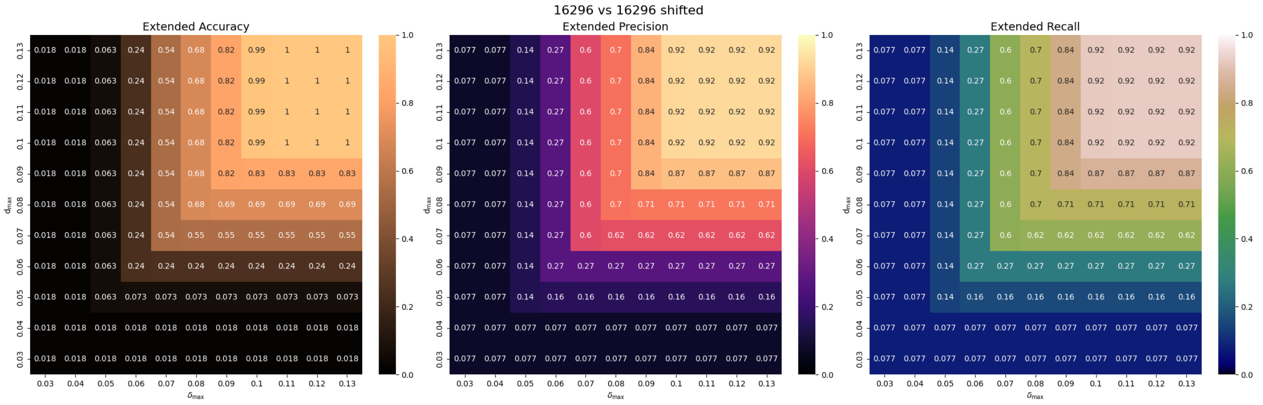

Supplementary Figure S43: Extended performance metrics computed for the alignment of the original 7D NMR spectrum of protein 16296 and the shifted 7D NMR spectrum of protein 16296.

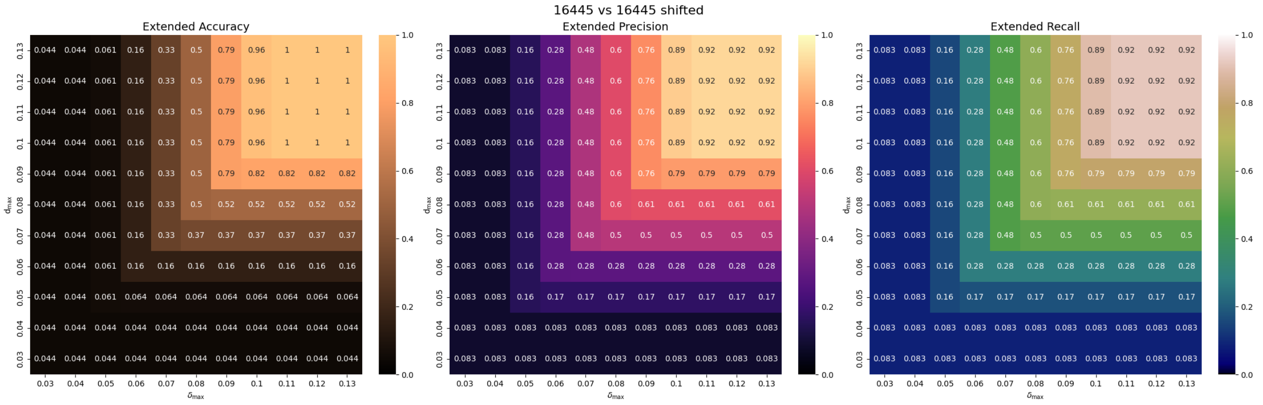

Supplementary Figure S44: Extended performance metrics computed for the alignment of the original 7D NMR spectrum of protein 16445 and the shifted 7D NMR spectrum of protein 16445.

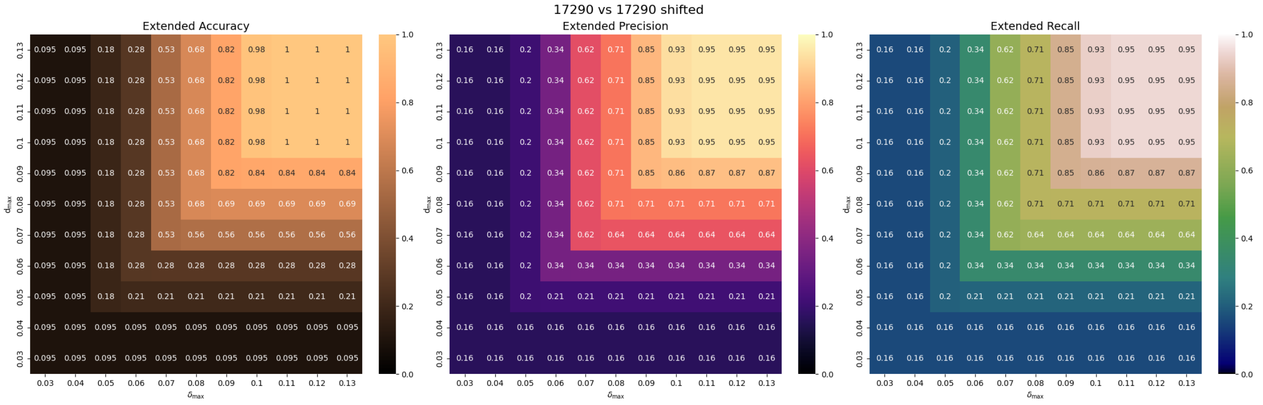

Supplementary Figure S45: Extended performance metrics computed for the alignment of the original 7D NMR spectrum of protein 17290 and the shifted 7D NMR spectrum of protein 17290.

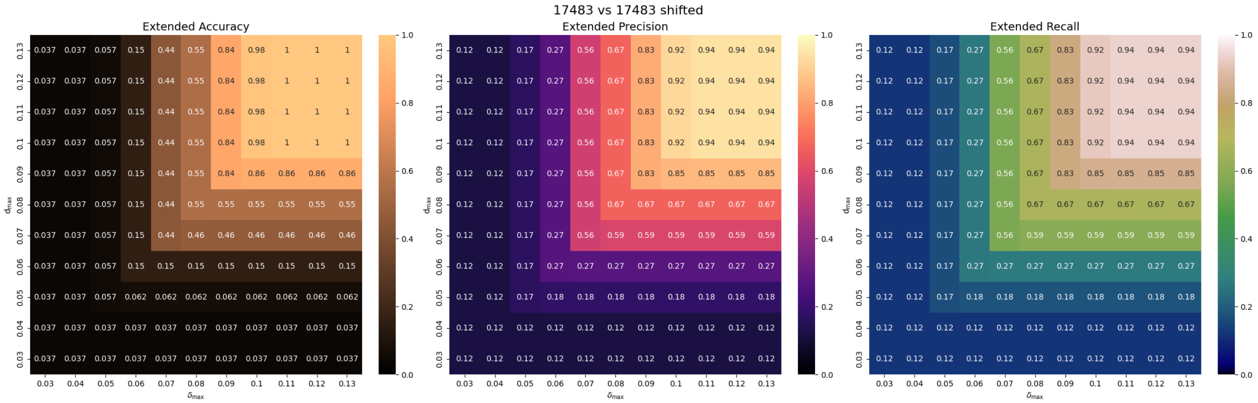

Supplementary Figure S46: Extended performance metrics computed for the alignment of the original 7D NMR spectrum of protein 17483 and the shifted 7D NMR spectrum of protein 17483.

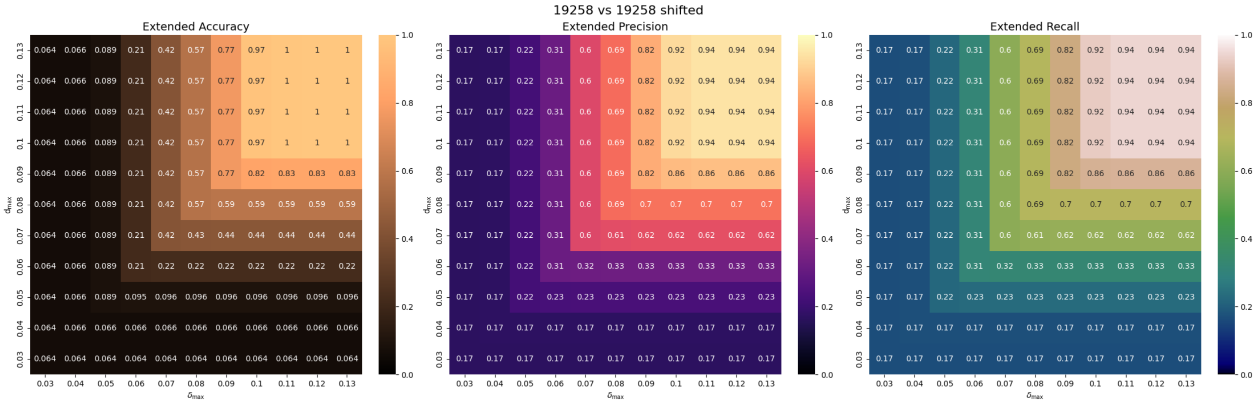

Supplementary Figure S47: Extended performance metrics computed for the alignment of the original 7D NMR spectrum of protein 19258 and the shifted 7D NMR spectrum of protein 19258.

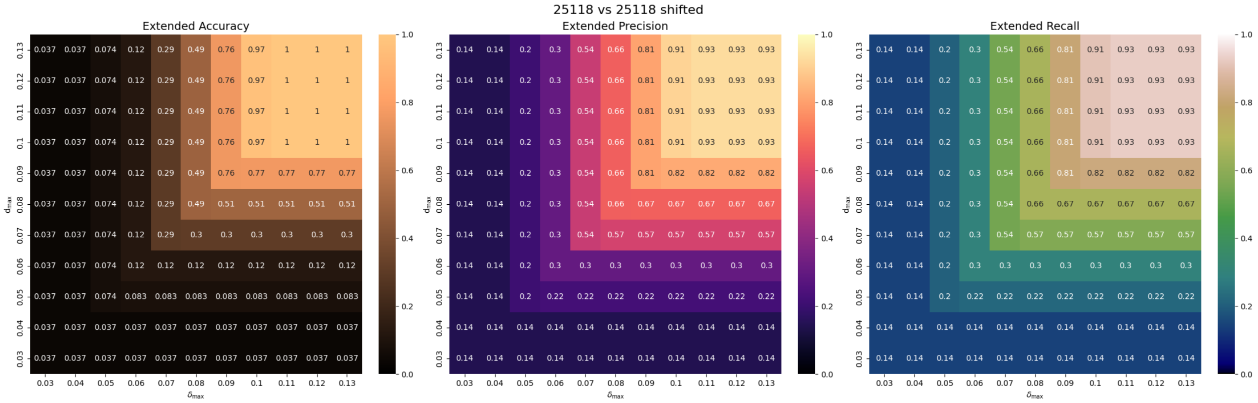

Supplementary Figure S48: Extended performance metrics computed for the alignment of the original 7D NMR spectrum of protein 25118 and the shifted 7D NMR spectrum of protein 25118.

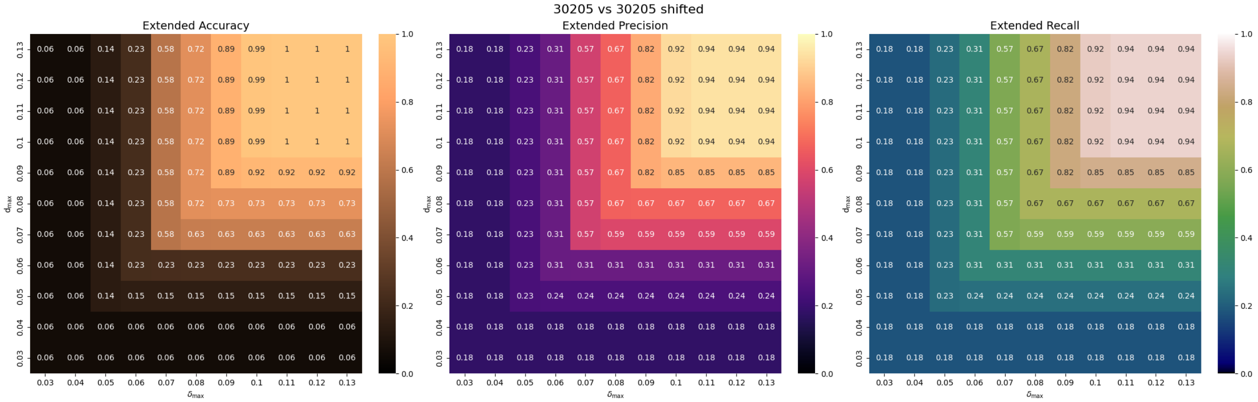

Supplementary Figure S49: Extended performance metrics computed for the alignment of the original 7D NMR spectrum of protein 30205 and the shifted 7D NMR spectrum of protein 30205.

References

[1] J. C. Hoch, K. Baskaran, H. Burr, J. Chin, H. Eghbalnia, T. Fujiwara, M. Gryk, T. Iwata, C. Kojima, G. Kurisu, D. Maziuk, Y. Miyanoiri, J. Wedell, C. Wilburn, H. Yao, and M. Yokochi. Biological Magnetic Resonance Data Bank. *Nucleic Acids Research*, 51(D1):D368–D376, Jan. 2023.

[2] P. Klukowski, F. F. Damberger, F. H.-T. Allain, H. Iwai, H. Kadavath, T. A. Ramelot, G. T. Montelione, R. Riek, and P. Güntert. The 100-protein NMR spectra dataset: A resource for biomolecular NMR data analysis. *Scientific Data*, 11(1):30, Jan. 2024. Publisher: Nature Publishing Group.

[3] E. Lange, R. Tautenhahn, S. Neumann, and C. Gröpl. Critical assessment of alignment procedures for lc-ms proteomics and metabolomics measurements. *BMC bioinformatics*, 9:375, 10 2008.

[4] J. A. Romero, P. Putko, M. Urbańczyk, K. Kazimierczuk, and A. Zawadzka-Kazimierczuk. Linear discriminant analysis reveals hidden patterns in NMR chemical shifts of intrinsically disordered proteins. *PLOS Computational Biology*, 18(10):e1010258, Oct. 2022. Publisher: Public Library of Science.

[5] G. Skoraczyński, A. Gambin, and B. Miasojedow. Alignstein: Optimal transport for improved LC-MS retention time alignment. *GigaScience*, 11:giac101, 11 2022.
